# Transcriptome analysis of the adult human Klinefelter testis and cellularity-matched controls reveals disturbed differentiation of Sertoli- and Leydig cells

**DOI:** 10.1038/s41419-018-0671-1

**Published:** 2018-05-22

**Authors:** Sofia Boeg Winge, Marlene Danner Dalgaard, Kirstine G Belling, Jacob Malte Jensen, John Erik Nielsen, Lise Aksglaede, Mikkel Heide Schierup, Søren Brunak, Niels Erik Skakkebæk, Anders Juul, Ewa Rajpert-De Meyts, Kristian Almstrup

**Affiliations:** 1 Department of Growth and Reproduction, Copenhagen University Hospital (Rigshospitalet), Copenhagen, Denmark; 2 DTU Multi-Assay Core, DTU Bioinformatics, Technical University of Denmark, Kongens Lyngby, Denmark; 3 Translational Disease Systems Biology Group, Novo Nordisk Foundation for Protein Research, University of Copenhagen, Copenhagen, Denmark; 4 Bioinformatics Research Center, Aarhus University, Aarhus, Denmark

## Abstract

The most common human sex chromosomal disorder is Klinefelter syndrome (KS; 47,XXY). Adult patients with KS display a diverse phenotype but are nearly always infertile, due to testicular degeneration at puberty. To identify mechanisms causing the selective destruction of the seminiferous epithelium, we performed RNA-sequencing of 24 fixed paraffin-embedded testicular tissue samples. Analysis of informative transcriptomes revealed 235 differentially expressed transcripts (DETs) in the adult KS testis showing enrichment of long non-coding RNAs, but surprisingly not of X-chromosomal transcripts. Comparison to 46,XY samples with complete spermatogenesis and Sertoli cell-only-syndrome allowed prediction of the cellular origin of 71 of the DETs. DACH2 and FAM9A were validated by immunohistochemistry and found to mark apparently undifferentiated somatic cell populations in the KS testes. Moreover, transcriptomes from fetal, pre-pubertal, and adult KS testes showed a limited overlap, indicating that different mechanisms are likely to operate at each developmental stage. Based on our data, we propose that testicular degeneration in men with KS is a consequence of germ cells loss initiated during early development in combination with disturbed maturation of Sertoli- and Leydig cells.

## Introduction

Klinefelter syndrome (KS) is caused by the presence of at least one additional X-chromosome in men. KS is the most common sex chromosomal disorder with an estimated prevalence of 1 in 660 newborn boys, but only one fourth of the expected cases are diagnosed throughout life^1^. There is a huge variation in the phenotype of men with KS. In adulthood, the classical symptoms are tall stature, gynecomastia, reduced virilization, hypergonadotropic hypogonadism, and small testes^2–5^. Many men with KS, however, first discover their condition when they want to have a child as the most pervasive symptom is azoospermia or, rarely, severe oligozoospermia.

The histology of a typical testis from an adult KS man shows absence of germ cells, high amounts of degenerated, hyalinized tubules, and Leydig cell hyperplasia^2,6^. In some men with KS, focal spermatogenesis is nevertheless observed and is considered the result of elimination of the supernumerary X-chromosome in a subset of spermatogonia^7–10^. Presence of focal spermatogenesis opens the possibility to recover sperm in the ejaculate or to perform surgical microdissection of the seminiferous tubules and isolate areas with focal spermatogenesis (micro-TESE procedures) that subsequently can be used for intracytoplasmic sperm injection. This allows such patients to become fathers of children with apparently normal karyotype (reviewed in refs. ^11^).

The molecular mechanisms responsible for the KS phenotype are poorly understood. Best described is the increased dosage of the X-chromosomal *SHOX* gene, which accounts at least in part for the tall stature observed in men with KS^12^. Recently, several studies have reported transcriptome analysis of peripheral blood samples^13–15^ and of cultured lymphocytes isolated from blood^16^ from KS patients compared to controls. The only overlap between the transcriptome profiles was upregulation of the long non-coding RNA (lncRNA) *XIST*^13–16^. Upregulation of *XIST* is expected and can be used as a quality control, as *XIST* is involved in X-chromosome inactivation (XCI)^17,18^. Only 12 transcripts were differentially expressed in more than one study^13,15,16^, highlighting the diverse phenotype.

Pre-pubertal testicular development in KS has been described as similar to boys with normal karyotypes, albeit with smaller testis volume and reduced germ cell numbers^6,19^. However, our recent study indicated that despite the supposedly normal pre-pubertal testicular development, the fetal KS testis displays impairment of fetal gonocyte differentiation resulting in loss of pre-spermatogonia^20^. This effect is most likely caused by the extra X-chromosome because the fetal KS testis transcriptome revealed enrichment of X-chromosomal transcripts as well as lncRNAs^20^. Later on, initiation of spermatogenesis at pubertal onset fails in the KS testis and results in testicular degeneration^6,19^.

Gene expression analysis of human KS testes is challenging, as available material is scarce because testicular biopsies are not performed in the routine clinical work-up. But three recent studies used leftover testis tissue from TESE and micro-TESE. In one study, expression of metabolic components was examined in six KS and six controls with azoospermia but conserved spermatogenesis^21^. In another study, D’Aurora et al. performed microarray analysis of bilateral testicular biopsies from six non-mosaic KS patients with absence of germ cells compared to a pool of three biopsies from patients with obstructive azoospermia but complete spermatogenesis^22^. While the material used in the two studies is unique and very precious, the huge cellularity differences are expected to drive most of the changes in the transcriptome and mask transcripts involved in the actual testicular phenotype of KS. In another study by D’Aurora et al., testicular biopsies from three atypical adult KS patients with focal spermatogenesis were compared to the controls used in their former study^23^. The cellularity differences were thereby less pronounced but the transcriptome now reflected the atypical situation of KS testis with focal spermatogenesis.

To describe the adult KS testicular transcriptome and to account for cellularity differences, we performed RNA-sequencing of fixed, paraffin-embedded testicular biopsies from adult KS patients and cellularity-matched controls (CMC), which had Sertoli-cell-only (SCO) pattern and Leydig cell hyperplasia. The cellular origin of the differentially expressed transcripts (DETs) was determined by including samples with complete spermatogenesis. Moreover, to describe developmental changes in the testicular KS transcriptome, we included testis samples from pre-pubertal KS boys along with our published data on the fetal KS testis.

## Results

### The adult KS testicular transcriptome

**Fig. 1:**
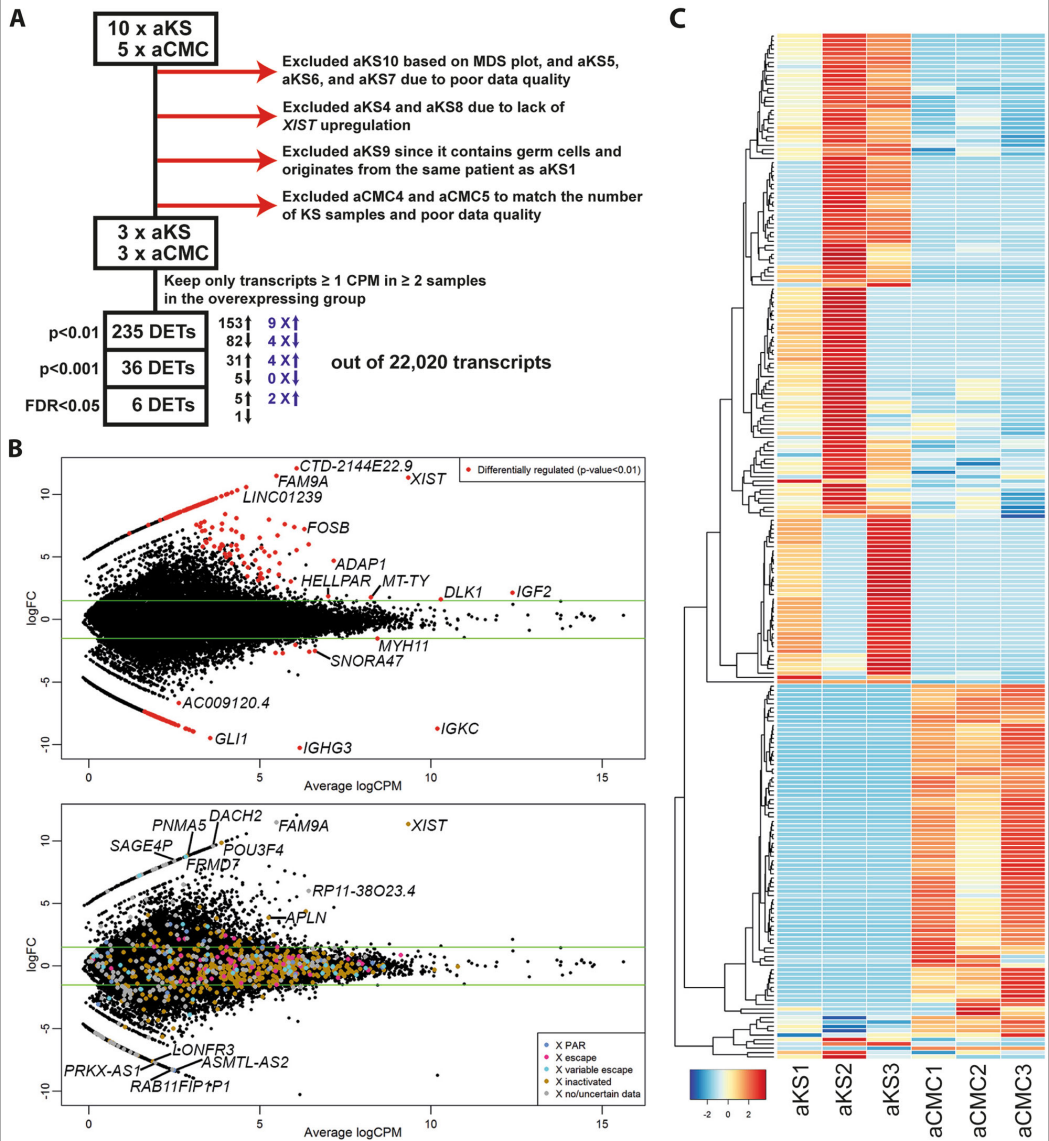
Differentially expressed transcripts (DETs) in testis from adult Klinefelter syndrome (KS) patients compared to cellularity-matched control (CMC) testes. **a** Flowchart depicting the exclusion of samples. **b** Top: Volcano plot with all expressed transcripts. The red dots are the DETs using an un-adjusted *p*-value < 0.01. The most significant DETs are indicated. Bottom: The same plot as on the top, but with all X-chromosomal transcripts plotted according to their X-chromosome inactivation status^25^. The significant X-chromosomal DETs are indicated. **c** Heatmap of the DETs

We performed RNA-sequencing of 15 fixed, paraffin-embedded testis samples, and the average reads per sample was 2.8 million (Table 1). The RNA quality was compromised and we, therefore, performed a thorough quality control based on MDS plots, heatmaps and expression of *XIST* in the KS samples (Fig. 1a and Supplementary Fig. S1). Based on this, three KS samples and three CMC were used in the subsequent analyses (Fig. 1a).

**Table 1:** Samples used for RNA-sequencing

ID	Group	Age	Karyotype (blood)	Fixative	Block age	Library size	Included in final analysis/ reason for exclusion
aKS1	Adult KS	21y, 7m	47,XXY	GR-fix	1 y	7,413,356	Yes
aKS2	Adult KS	27y, 7m	47,XXY	Bouin	14 y	1,693,175	Yes
aKS3	Adult KS	28 y, 7 m	47,XXY	GR-fix	4 y	2,199,570	Yes
aKS4^a^	Adult KS	30 y, 7 m		Stieve	42 y	812,877	No *XIST* expression
aKS5	Adult KS	40y, 2m	47,XXY	Stieve	16 y	177,280	Low library size and MDS plot
aKS6	Adult KS	21y, 3m	48,XXYY, 47,XXY	Stieve	40 y	132,405	Low library size and MDS plot
aKS7^a^	Adult KS	27 y, 11 m		Stieve	48 y	541,108	MDS plot
aKS8^a^	Adult KS	28 y, 6 m		Stieve	48 y	123,653	No *XIST* expression
aKS9	Adult KS, GCs	21y, 7m	47,XXY	GR-fix	1 y	8,233,082	Same patient as aKS1
aKS10	Adult KS, GCs	30y, 0m	47,XXY	Stieve	14 y	188,313	Low library size and MDS plot
aCMC1	Adult CMC	32 y, 0 m	46,XY	GR-fix	6 y	4,480,564	Yes
aCMC2	Adult CMC	32y, 7m	Two *SHOX* copies	GR-fix	4 y	7,868,360	Yes
aCMC3	Adult CMC	33 y, 8 m	46,XY	GR-fix	7 y	2,044,132	Yes
aCMC4	Adult CMC	39 y, 8 m	46,XY	GR-fix	5 y	3,113,958	Balance with number of KS samples
aCMC5	Adult CMC	42 y, 2 m	46,XY	GR-fix	7 y	2,443,579	Balance with number of KS samples
aSCO1	Adult SCO	29 y, 6 m	46,XY	GR-fix	3 y	3,064,063	
aSCO2	Adult SCO	31 y, 7 m	46,XY	GR-fix	7 y	2,317,076	
aSCO3	Adult SCO	33 y, 3 m	46,XY	GR-fix	6 y	7,157,424	
aSCO4	Adult SCO	35 y, 4 m	46,XY	GR-fix	6 y	3,897,228	
aSCO5	Adult SCO	43 y, 5 m	46,XY	GR-fix	5 y	634,570	Low library size and MDS plot
aNorm1^b^	Adult NT	30 y, 2 m	46,XY^d^	GR-fix	4 y	4,635,540	Yes
aNorm2^c^	Adult NT	31 y, 3 m	46,XY^e^	GR-fix	6 y	3,100,541	Yes
aNorm3^c^	Adult NT	35 y, 9 m	46,XY^e^	GR-fix	6 y	1,213,352	Yes
aNorm4^b^	Adult NT	39 y, 9 m	No karyotype^d^	GR-fix	5 y	373,341	Yes
pKS1^a,d^	Pre-pubertal KS	9 y, 6 m		Stieve	39 y	442,161	Yes
pKS2^a,d^	Pre-pubertal KS	9 y, 6 m		Stieve	35 y	481,952	Yes
pKS3^a,d^	Pre-pubertal KS	11 y, 0 m		Cleland	32 y	404,698	Yes
pKS4^a,d^	Pre-pubertal KS	14 y, 1 m		Stieve	37 y	231,918	Yes
pKS5^a,d^	Pre-pubertal KS	14 y, 9 m		Stieve	39 y	1,120,193	No *XIST* expression
pNorm1^e^	Pre-pubertal NT	7 y, 5 m		Stieve	21 y	671,897	Yes
pNorm2^f^	Pre-pubertal NT	7 y, 9 m		Cleland	30 y	1,026,282	Yes
pNorm3^f^	Pre-pubertal NT	10 y, 2 m		Cleland	30 y	392,993	Yes
pNorm4^f^	Pre-pubertal NT	10 y, 10 m		Stieve	35 y	268,552	Yes

*KS* Klinefelter syndrome, *GCs* germ cells, *SCO* Sertoli cell-only, *NT* normal testis (biopsy with complete spermatogenesis), *GR-fix* modified Stieve’s fixative, *y* years, *m* months

^a^Known KS patient, no karyotype available

^b^Testis cancer patient

^c^Man from infertile couple

^d^Biopsy taken due to suspicion of urological problems

^e^Acute lymphocytic leukemia patient

^f^Autopsy

Using an un-adjusted *p*-value < 0.01, 235 DETs were identified, with 153 being upregulated and 82 being downregulated in KS compared to CMC (Fig. 1b, c, and Supplementary Tables S1 and S2). The most significant DET was *XIST*, as expected. Apart from *XIST*, eight X-chromosomal DETs were upregulated (Fig. 1b and Supplementary Table S1); three of these, *POU3F4*, *PNMA5*, and *ALPN*, have previously been reported to undergo XCI^24^. The remaining upregulated transcripts have no or discordant data regarding XCI status. Four X-chromosomal transcripts were downregulated, one expressed from PAR1: *ASMTL-AS1*, one previously reported to undergo XCI: *LONRF3*, and two with no or discordant data regarding XCI (Fig. 1b and Supplementary Table S2).

### Protein expression of DACH2 and FAM9A

To validate our findings, we selected two upregulated X-chromosomal candidates, *DACH2* and *FAM9A* based on availability of antibodies and supportive literature.

**Fig. 2:**
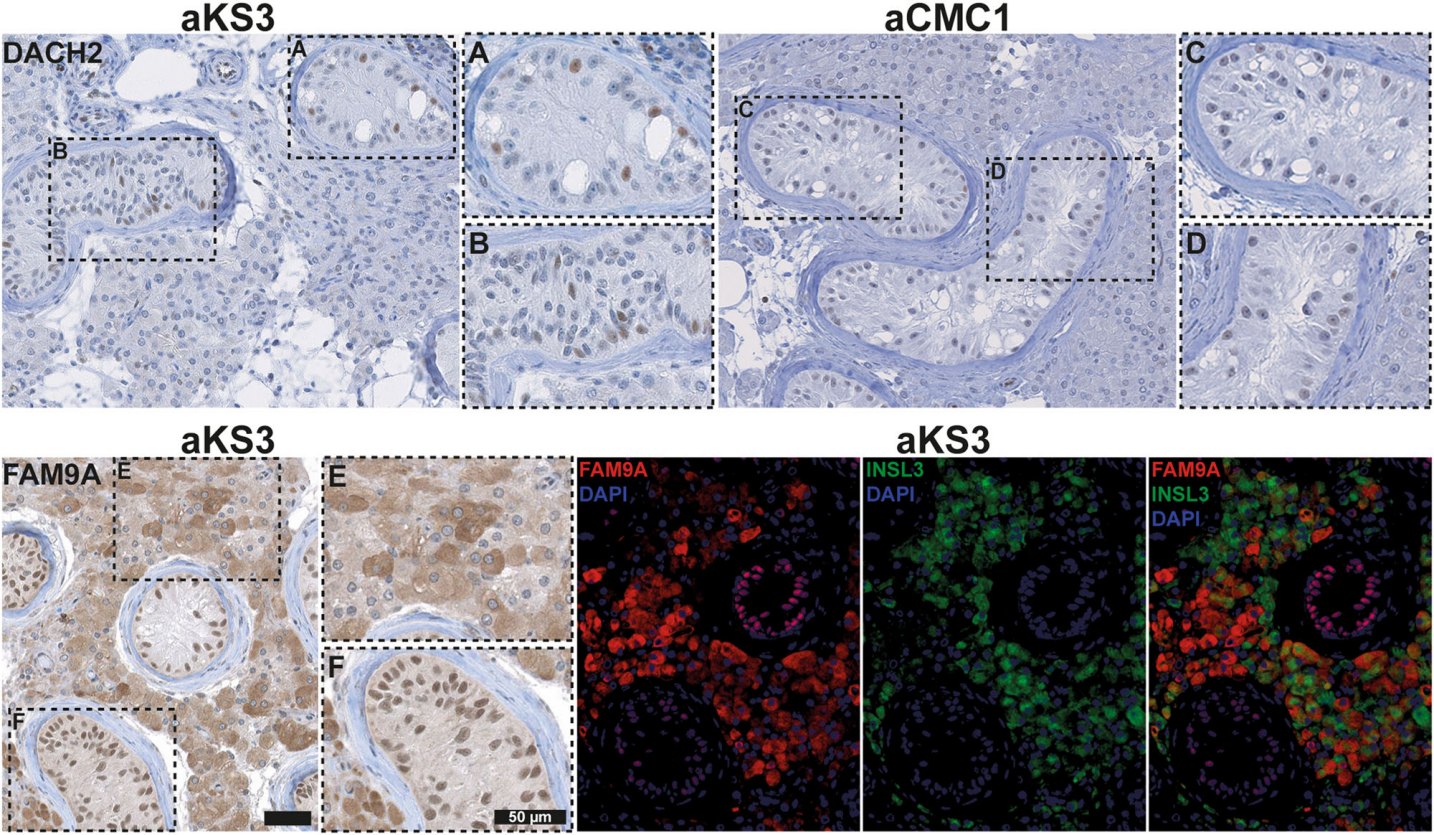
Localization of DACH2 and FAM9A in a testis from an adult man with Klinefelter syndrome (KS). IHC staining with antibodies against DACH2 and FAM9A of a testis from an adult man with KS (which is the same sample as aKS3 used for RNA-sequencing). DACH2 was expressed in a fraction of nuclei both in type A (mature) Sertoli cell-only (SCO) tubules (**a**) and type B (immature) SCO tubules (**b**). The same pattern was seen in another KS testis, aKS1, also used for the transcriptome profiles (data not shown). In the CMC testis (*n* = 3) (exemplified here aCMC1 used for RNA-sequencing), DACH2 was only expressed weakly (**c**, **d**) In adult KS, FAM9A was expressed in Leydig cells with varying intensity (**e**) and in all Sertoli cell nuclei (**f**). Immunofluorescence staining showed that FAM9A and INSL3 were expressed by two populations of Leydig cells with limited overlap. Scale bars correspond to 50 µm

Immunohistochemical staining showed that DACH2 was expressed in the nuclei. In testes with complete spermatogenesis, DACH2 showed strongest intensity in the wall of blood vessels, medium-strong intensity in primary spermatocytes, and faint-negative staining in Sertoli cells (Supplementary Fig. S2). In testes from adult men with KS (*n* = 2), DACH2 was expressed in a subset of Sertoli cell nuclei of type A (mature) and type B (immature) SCO tubules (identified according to ref. ^25^) (Fig. 2a, b). Sertoli cell nuclei showed only faint DACH2 staining in CMC biopsies (*n* = 3) (Fig. 2c, d), which confirmed upregulation of DACH2. To test whether DACH2 expression depended on the Sertoli cell maturation stage, an adjacent section was stained with AMH, a marker of immature Sertoli cells. In Sertoli cells, AMH was expressed in the cytoplasm with varying intensity, with the majority of DACH2-positive Sertoli cells also positive for AMH (Supplementary Fig. S3). However, a subset of AMH-positive Sertoli cells were negative for DACH2, and a small proportion of the DACH2-positive Sertoli cells were negative for AMH (Supplementary Fig. S3).

In a fetal testis aged gestational week (Gw)10, a large fraction of the Sertoli cells indeed showed expression of DACH2 (Supplementary Fig. S2).

In testes with complete spermatogenesis, FAM9A showed strongest intensity in the cell membrane of spermatocytes and spermatids, moderate-strong intensity in Sertoli cell nuclei and faint-strong staining in Leydig cell cytoplasm (Supplementary Fig. S2). In the testis from an adult KS patient, FAM9A was expressed in the cytoplasm of Leydig cells with varying intensity and in the nuclei of all Sertoli cells (Fig. 2e, f), and the same pattern was seen in CMC testes (*n* = 3) (data not shown). A double fluorescent staining with FAM9A and insulin-like 3 (INSL3, a marker of mature Leydig cells^26^) showed that FAM9A and INSL3 were only partially overlapping with distinct FAM9A-positive/INSL3-negative and FAM9A-negative/INSL3-positive populations visible (Fig. 2), indicating that FAM9A is expressed predominantly in immature Leydig cells and during the transition phase into mature Leydig cells. In a fetal testis aged Gw10, a fraction of interstitial cells (most probably fetal Leydig cells) were positive for FAM9A (Supplementary Fig. S2).

### Enrichment analyses of the KS DETs

**Fig. 3:**
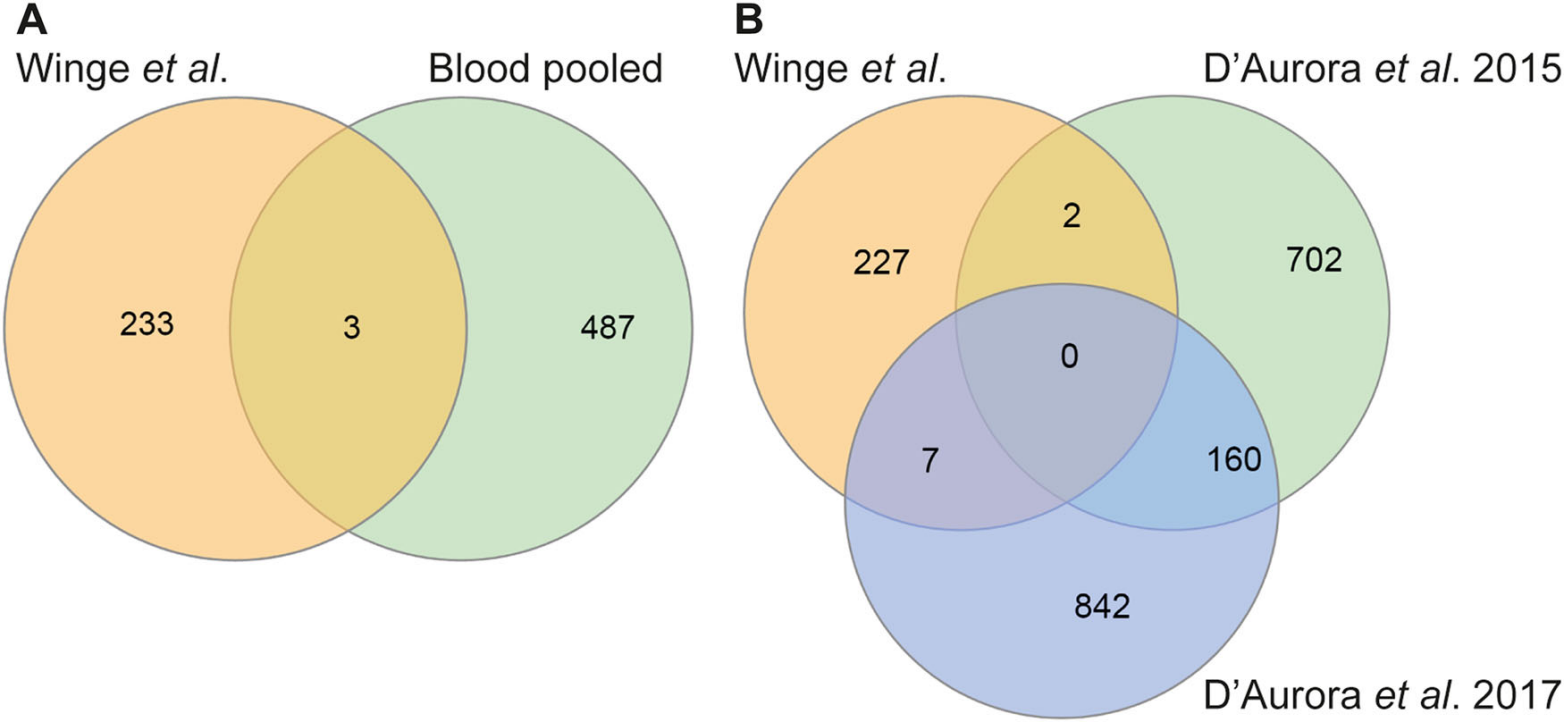
Overlap of the differentially expressed transcripts (DETs) with previous studies. Overlap with previous studies performed on blood^14–17^ (**a**) and on testis tissue^23,24^ (**b**). The overlapping transcripts can be seen in Supplementary Table S3

Next, we compared DETs from previous studies of KS patients^13–16,22,23^, and found an overlap of three DETs with the transcriptome profiles from blood and nine DETs from the studies on testes (Fig. 3a, b). Overlapping DETs included *FOSB* and *DLK1* (Supplementary Table S3).

**Fig. 4:**
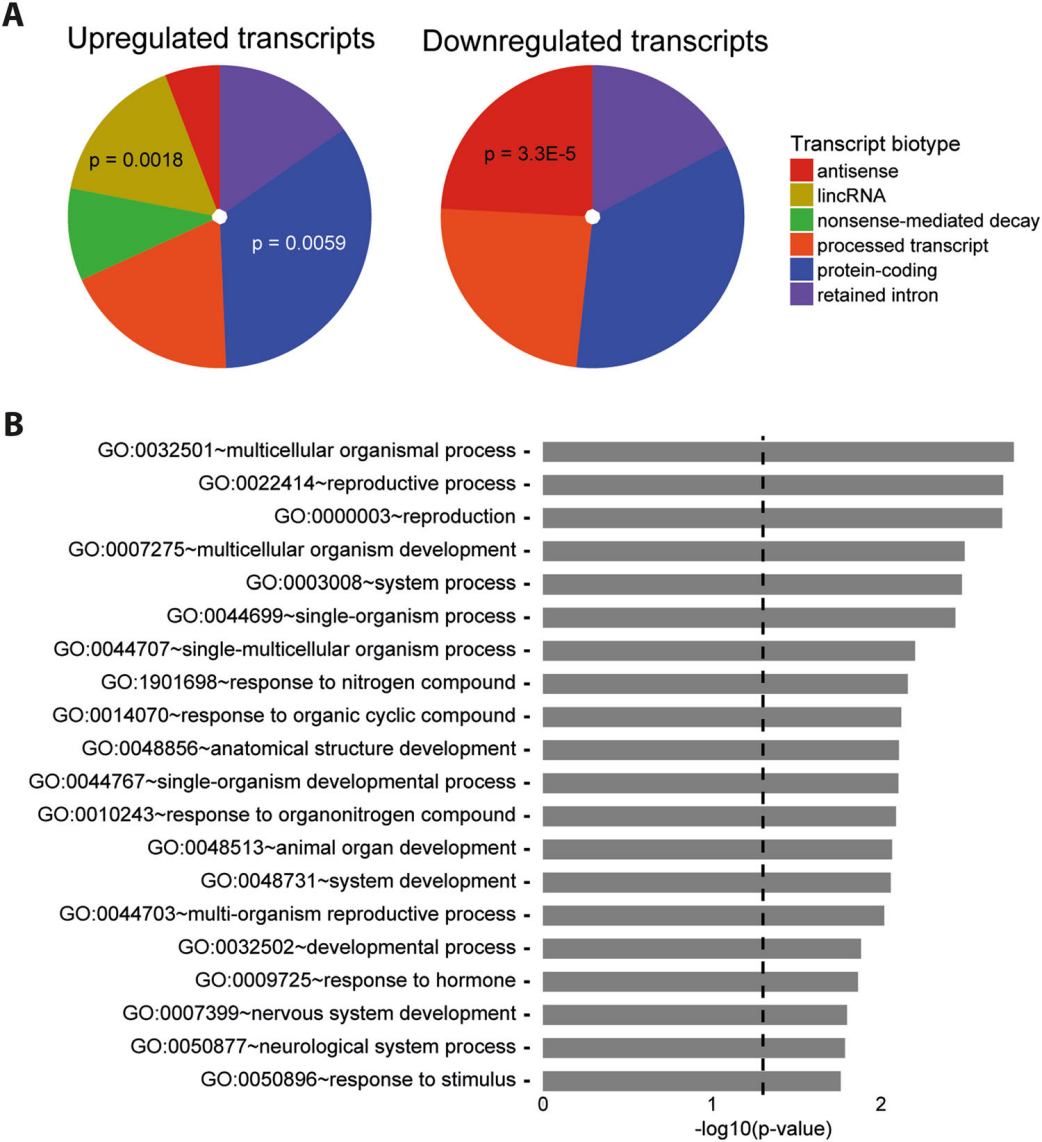
Enrichment analyses of the differentially expressed transcripts (DETs). **a** Distribution in RNA biotype among the upregulated (left) and downregulated (right) transcripts. The significant biotypes are indicated with *p*-values. For RNA biotypes of each individual DET, see Supplementary Tables S1 and S2, and for description of RNA biotypes, see Supplementary Table S4. **b** Significantly overrepresented gene ontology (GO) “Biological Process” terms of the DETs

We noticed that many of the DETs were ncRNAs (Supplementary Tables S1 and S2), and we, therefore, analyzed for enrichment of transcript biotypes classified by the GENCODE annotation^27^. We found significant (*p*-value = 0.0018) enrichment of long, intergenic ncRNAs (lincRNAs) among the upregulated transcripts. Also, protein-coding transcripts among the upregulated (*p*-value = 0.0058) and antisense transcripts among the downregulated transcripts were significantly enriched (*p*-value = 3.3E-5) (Fig. 4a and Supplementary Table S4).

To gain insight into the potential function of the DETs, we searched for enrichment of particular gene ontologies (GO). Among the most interesting results were those involved in reproduction (“reproductive process”, “reproduction”, and “multi-organism reproductive process”), development (“multi-cellular organism development”, “single-organism developmental process”, “animal organ development”, “system development”, and “developmental process”), as well as “response to hormone” (Fig. 4b).

### Cellular origin of the KS DETs

In order to identify the potential cellular origin of the 235 DETs, we used a comparative approach, and profiled the transcriptome of five adult testis biopsies with SCO and normal amounts of Leydig cells, and four adult testis biopsies with full spermatogenesis and normal amounts of Leydig cells (these samples are termed Norm) (Table 1 and Supplementary Fig. S4). We excluded one SCO sample due to poor quality (data not shown).

**Fig. 5:**
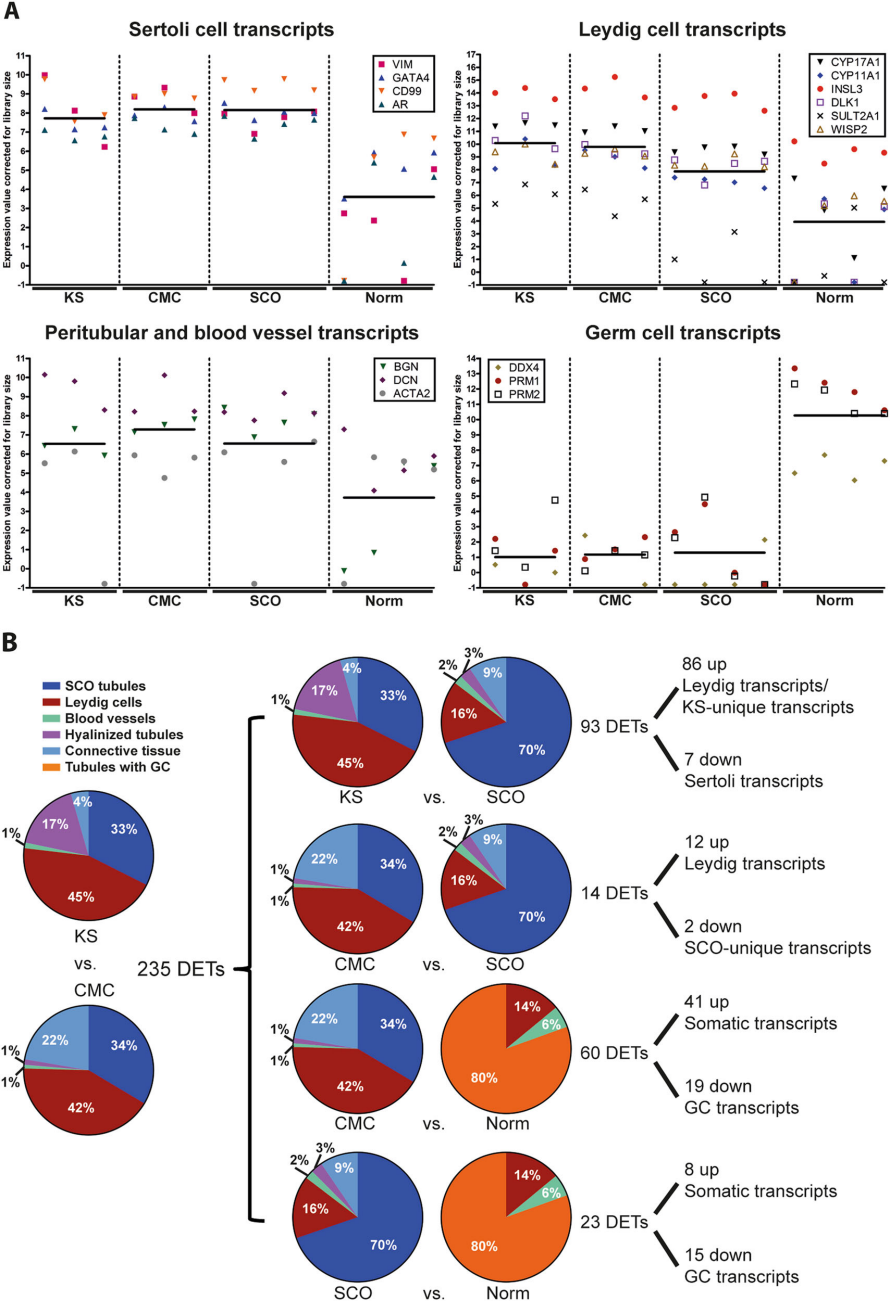
Depicted cellular origin of the differentially expressed transcripts (DETs). **a** Known Sertoli, Leydig, peritubular/blood vessel and germ cell markers expressed in the testis of Klinefelter syndrome (KS), cellularity-matched controls (CMC), Sertoli cell-only (SCO), and normal testes with complete spermatogenesis (Norm). The lines indicate the mean expression values in each group. **b** Pie charts of average cellularity in each group and resultant predicted cellular origin of the overlapping DETs taking into account the amounts of cellularity transcripts in (**a**)

First, we compared the expression of known cell type-specific transcripts in the four groups (Fig. 5a). As expected, KS and CMC specimens displayed relative upregulation of Leydig cell transcripts compared to SCO samples, which had a higher level than the Norm samples. The same tendency was seen for peritubular/blood vessel transcripts. The levels of Sertoli cell transcripts were similar among KS, CMC, and SCO, but lower in the Norm samples. And as expected, the levels of germ cell transcripts were highest in Norm samples (Fig. 5a).

**Fig. 6:**
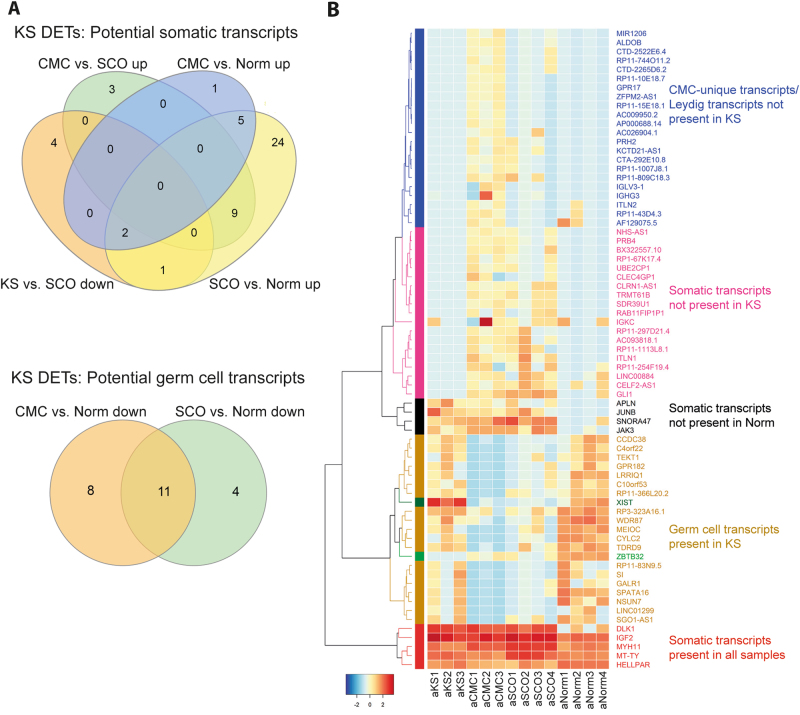
Expression of transcripts with depicted cellular origin in each sample. **a** Venn plots of the transcripts from the comparisons in Fig. 4b. The upregulated transcripts in KS vs. SCO and the downregulated transcripts in CMC vs. SCO are not included as they are predicted to be unique for KS and SCO. **b** Heatmap of the transcripts from (**a**). The clustering is made so that the different cellularity transcripts are grouped together

We also evaluated the cellularity of each sample by measuring the area of the cellular compartments (Supplementary Fig. S4) and when calculating the average cellularity, we could predict the cellular origin of the DETs for each comparison (Figs. 5b and 6a).

We compared the DETs with those of all of the group comparisons, and thereby could get an idea about the cellular origin of the individual transcripts. There was some overlap between the comparisons, allowing prediction of cellular origin of 71 out of the 235 DETs (Fig. 6a, b). Twenty-six were upregulated in KS and the remaining 45 were downregulated. We were not able to predict the cellular origin of 127 of the upregulated transcripts and 37 of the downregulated transcripts, which could be because the transcripts are expressed in many different cell types or that they are only expressed in the KS testis.

### Overlap between DETs in the KS testis throughout development

In our previous study^20^, we identified 211 DETs in the fetal KS testis compared to controls. To investigate changes of the KS testis transcriptome throughout development, we performed transcriptome analysis of testis samples from pre-pubertal KS boys and age-matched controls (Supplementary Fig. S5). The data quality was severely compromised with on average 490,000 reads per sample (Table 1). We did sanity checks, and one of the pre-pubertal KS samples did not have expression of *XIST* and was excluded (Supplementary Fig. S6). We identified 181 DETs, with 139 being downregulated (Supplementary Fig. S7 and Supplementary Tables S5 and S6).

**Fig. 7:**
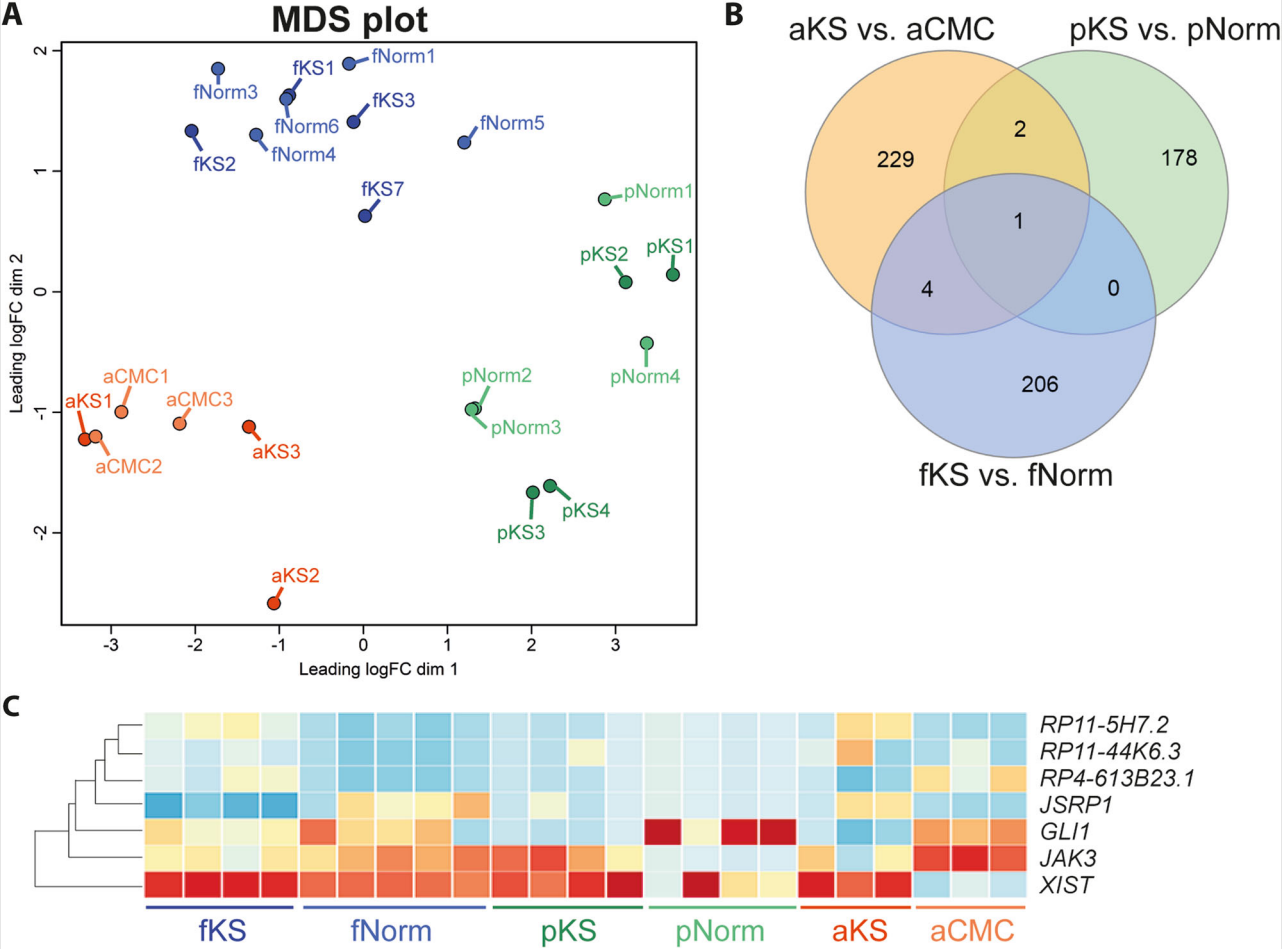
Overlap in adult Klinefelter syndrome (KS) differentially expressed transcripts (DETs) with fetal and pre-pubertal DETs. **a** MDS plot showing that the different age groups are separated from each other, rather than KS and controls. **b** Venn plot showing the overlap with the DETs of fetal^21^, pre-pubertal and adult KS. **c** Heatmap showing the expression level of the overlapping transcripts in each developmental group. More details can be found in Supplementary Table S7

When we compared the testicular transcriptome profiles at the three different developmental time points in an MDS plot, each age group clustered separately, rather than clustering according to the karyotype (Fig. 7a). Only seven transcripts overlapped between the three developmental time points (Fig. 7b, c, and Supplementary Table S7). Of these, only *XIST* was shared at all time points. Apart from *XIST*, none of overlapping DETs were expressed from the X-chromosome. Two transcripts were upregulated in fetal and adult KS (*RP11-15H7.2* and *RP11-44K6.3*). One transcript was downregulated in pre-pubertal and adult KS (*GLI1*), and the remaining transcripts showed different directions of regulation between two time points (Fig. 7c).

## Discussion

It is intriguing that an extra X-chromosome in KS patients is associated with loss of germ cells, although the adult testicular phenotype with degeneration and hyalinization of seminiferous tubules does not develop until puberty. It is to be expected that chromosome pairing and segregation during meiosis is impaired with the presence of an extra chromosome. However, since the loss of spermatogonia begins before puberty, even as early as during fetal development, other mechanisms must be involved. To bring new insights into the functional mechanisms that result in germ cell loss and testicular degeneration we analyzed the testicular transcriptome. It was admittedly challenging to obtain informative transcriptome profiles from the archived material. Furthermore, tubular and interstitial compartments of the testis contain many different types of cells. In order to tackle this, we collected all the testicular biopsies from KS men that were available in our archives. However, of the 12 samples available, only 10 could be used for RNA-sequencing and only 3 KS samples were found suitable in the analysis. Moreover, we did not have statistical power to correct for multiple testing, which is normal practice in transcriptome analysis. Instead, we used an un-adjusted *p*-value and identified 235 DETs potentially involved in the pathogenesis of the KS testicular phenotype.

One of the interesting DETs was *DACH2*, with a potential function in “reproduction” and “development” according to the GO analysis. The potential involvement of DACH2 in the testicular phenotype of KS men is supported by two studies. One study showed that *Dach2* was expressed in the fetal mouse testis, but not in the ovary, with pronounced expression in interstitial cells. Interestingly, the fetal gonadal expression of *Dach2* was lost in the Wnt-4 knock-out testis^28^. Another study identified an association between several rare mutations in the *DACH2* gene and premature ovarian failure, which is characterized by an early loss of germ cells^29^. We validated the protein expression of DACH2 in the adult KS testis, and observed an interesting staining pattern. DACH2 was specifically expressed in a subset of Sertoli cell nuclei in the KS testis, which was not observed in the CMC. This indicates that DACH2 is a marker of immature Sertoli cells in the adult KS testis, which was supported by identification of DACH2 in fetal Sertoli cells and by the majority of DACH2-positive Sertoli cells in the adult KS testis being positive for AMH. Further studies are needed to corroborate this finding.

Interestingly, FAM9A, also appeared to be linked to the somatic cell maturation stage. FAM9A was expressed in a subset of Leydig cells, which appeared to be less mature than normal adult Leydig cells, which express INSL3^26^, and FAM9A was also identified in fetal Leydig cells. FAM9A was, in addition, expressed in all nuclei of Sertoli cells, but not at the fetal stage. The difference in the subcellular localization between Leydig- and Sertoli cells indicates that FAM9A has different functions in the two cell types. Nevertheless, both DACH2 and FAM9A seem to mark less mature somatic cell types in the KS confirming previous observations of immature Sertoli- and Leydig cells in the adult KS testis^25,26^. This hypothesis is further supported by the enrichment of GO terms, which revealed overrepresentation of terms related to “reproduction”, “development”, and “response to hormone”. Dysregulation of these pathways in general fits very well with the KS phenotype.

Another interesting upregulated KS transcript was *PNMA5*, which recently was shown to induce apoptosis^30^. According to the GTex portal (http://www.gtexportal.org/home/gene/PNMA5, accessed 7 Sept 2017). and the Human Protein Atlas (http://www.proteinatlas.org/ENSG00000198883-PNMA5/tissue, accessed 7 Sept 2017), PNMA is expressed exclusively in the testis and brain. Yet another pro-apoptotic protein upregulated in the KS samples was AIFM3^31^. Increased apoptosis could be a very relevant mechanism responsible for degeneration of the KS testis but when KS samples were stained with cleaved PARP (cPARP), a known marker of apoptotic cells, we only observed very few positively stained cells (data not shown).

When we compared our 235 KS DETs with previous transcriptome studies of KS samples, the overlap consisted of only 12 transcripts. Three DETs, were identified in studies performed on peripheral blood^13–16^ and the remaining nine were identified in studies performed on adult testis biopsies^22,23^. Since the control used in the testicular studies^22,23^ included complete spermatogenesis, the overlap with our study, using CMC, was expected to be limited. Moreover, the microarray approach used in these studies would not detect all types of transcripts (e.g., lncRNAs) contrary to the RNA-sequencing approach we used. In line with our previous study of fetal KS samples^20^, we indeed observed an enrichment of lincRNAs among the upregulated transcripts in the adult KS testis. This indicates that dysregulation of lincRNAs may play a central role in the KS testis.

Among the protein-coding DETs, three overlapped with previous studies; *DLK1*, *FAM9A* and *FBOXO43*, which all have been described previously in relation to testicular function. In the mouse, *Fbxo43* is expressed solely in the testis and ovary^32^, and was shown to be involved in meiosis I progression in rodent spermatocytes^33,34^. DLK1 is a marker of immature Leydig cells^26^ and its upregulation detected in the previous study^23^ and in our present study is consistent with the presence of more immature Leydig cells in the KS testis. In line with this, expression of *FAM9A*, which has been shown to be testis-specific^35^, was also found in less mature Leydig cells in our present study. Taken together, these results indicate that the somatic compartment in the adult KS testis is less mature than that in CMC.

When comparing the KS DETs in fetal, pre-pubertal and adult testes, the identified overlap was limited, indicating that different mechanisms are responsible for the testicular architecture and cellular composition at the different developmental stages. We did see an overlap in dysregulation of *JAK3* and *GLI1*. *JAK3* was upregulated in the pre-pubertal samples but downregulated in the adult samples, and *GLI1* was downregulated both in pre-pubertal and adult samples. A relationship between GLI1 and JAK3 was identified in acute T-cell lymphoblastic leukemia, where their expression levels showed a positive correlation^36^. As GLI1 is a component of Hedgehog signaling, which regulates development and cancer progression^37^, downregulation of this transcript in both pre-pubertal and adult samples, as well as downregulation of *JAK3* in the adult samples, may indicate a disturbance in the development of the KS testis. Indeed, Hedgehog signaling within the primary cilia of Sertoli cells from adult KS samples has previously been described to be different to that of Sertoli cells in tubules with ongoing spermatogenesis^38^. In our previous study of the fetal KS testis transcriptome^20^, we found enrichment of X-chromosomal DETs, which we could not recapitulate in the adult (or pre-pubertal) KS testis. This indicates that the effect of the extra X-chromosome is more direct in fetal life than in the adult. The adult KS testis consequently seems a *consequence* of the disturbed fetal gonadal development *caused* directly by the extra X-chromosome.

Based on our results we hypothesize that the overexpressed genes from the extra X-chromosome in patients with KS are directly causing changes in the testicular transcriptome primarily in fetal life, leading to the initiation of the germ cell loss, gonadal dysgenesis, and subsequent failure of the differentiation of the somatic cells, which consequently appears immature in the adult KS testis. Clearly our study was limited by lack of power due to compromised quality of the isolated RNA. Hopefully, technical advancements in the field will allow future studies to isolate RNA of better quality. Future studies should also focus on including more samples, as the individual variation in testicular development besides variations in cellularity is quite substantial. The present study nevertheless provided reference data for further mechanistic studies, and could also have some clinical implications concerning management of patients with KS. Our results indicate that intervention by supporting or stimulating development of the testicular somatic niche already during childhood could increase chances of fertility in adulthood. Before puberty, many boys with KS still have germ cells in the testis that with proper support by somatic cells might enter spermatogenesis. Such interventions, however, need to be investigated before they can be considered.

In conclusion, we were able to analyze the transcriptome of a total of 24 adult KS testis and controls. Enrichment analysis and protein expression patterns showed disturbed maturation of Sertoli-  and Leydig cells. We identified enrichment of lincRNAs, but not of X-chromosomal transcripts, indicating an indirect effect of the supernumerary X-chromosome. Furthermore, the adult testicular KS transcriptome showed a very limited overlap with that of pre-pubertal and fetal KS testis, indicating that distinct processes occur at these stages of development. Our study indicates that Sertoli-  and Leydig cells in the testis of adult men with KS are immature and that impairment of testicular function has its origin early in development.

## Materials and methods

### Tissue samples

The use of testicular tissue for this project was approved by a regional medical and research ethics committee (permit no. H-2-2014-103) and the Danish Data Protection Agency (no. 2012-58-0004, local no. 30-1482, I-Suite 03696). The testis samples were collected between 1976 and 2014. Following surgical excision, the samples were immediately fixed in different fixatives, dehydrated, and embedded in paraffin (Table 1). The blocks were stored either at +4 °C or −20 °C until sectioning.

### Immunohistochemistry (IHC)

Details on antibodies and detection reagents are shown in Supplementary Table S8.

IHC staining with anti-MAGE-A4 and anti-cPARP antibodies was performed essentially as described before^39^. For MAGE-A4, staining was performed on testes from ten adult men with KS, five with CMC testicular histology, five with Sertoli-cell-only (SCO), and four with full spermatogenesis (Table 1). In brief, paraffin-embedded sections were deparaffinized and rehydrated. Antigen retrieval was accomplished by microwaving the sections for 15 min in unmasking buffer. Then sections were incubated with 2% non-immune goat serum (Zymed Histostain Kit, Life Technologies, CA, USA) to minimize cross-reactivity. The primary antibody was added and incubated overnight at +4 °C, and then the sections were incubated with biotinylated goat anti-mouse IgG (MAGE-A4) or biotinylated goat-anti-rabbit (cPARP), before a peroxidase-conjugated streptavidin complex was used as a tertiary layer (Zymed Histostain Kit). Visualization was performed with amino ethyl carbasole (Zymed Histostain Kit).

Staining with anti-DACH2 and anti-FAM9A were performed on testes from two adult men with KS, three with full spermatogenesis and three with CMC testicular histology, and staining with anti-AMH was performed on testes from two adult men with KS (Supplementary Table S9), essentially as described before^40^. In brief, sections were subjected to pressure cooking-induced antigen retrieval in unmasking buffer at 110 °C for 30 min. Unspecific binding was blocked with 0.5% skimmed milk in TBS. Sections were incubated overnight with primary antibody diluted in skimmed milk at +4 °C. Horse anti-rabbit IgG HRP (ImmPRESS detection kit, Vector laboratories, CA, USA) was applied for 30 min followed by development with diaminobenzidin DAB (ImmPress detection kit) and nuclear counterstaining with Mayer’s Hematoxylin. Control sections with omission of primary antibody were all negative (data not shown).

### Double immunofluorescence

Details of antibodies and detection reagents are shown in Supplementary Table S8. Staining was performed on a testis sample from an adult man with KS (Supplementary Table S9) essentially as described before^40^. In brief, following pressure cooking treatment as described for IHC, the section was incubated with horse serum diluted in PBS containing 5% (w/v) BSA. The anti-FAM9A antibody was applied and the slides were left overnight at +4 °C and 1 h at room temperature. The section was incubated with peroxidase-conjugated chicken anti-rabbit secondary antibody (Sigma-Aldrich, MO, USA) for 30 min followed by incubation with Tyramide Cy3 (Perkin Elmer, MA, USA) for 10 min. Antigen retrieval with pressure cooker, blockade with horse serum, and incubation with primary antibody was repeated for the anti-INSL3 antibody. The section was then incubated with peroxidase-conjugated chicken anti-rabbit secondary antibody followed by incubation with Tyramide Fluorescien (Perkin Elmer) for 10 min. Finally, the section was counterstained with DAPI (Sigma-Aldrich). The section was imaged with an Olympus BX61 microscope, captured using the Cell Sense Dimensions V1.6 software (Olympus Ltd., Ballerup, Denmark) and processed in Adobe Photoshop 6.0.

### Quantification of cellularity

Adobe Photoshop CC 2014 (Adobe Systems Inc, CA, USA) was used to assess the cellularity of each sample. The area of tubules with germ cells was evaluated on the basis of the MAGE-A4-staining and was quantified with the “lasso” tool. SCO tubules, areas of Leydig cells, blood vessels, hyalinized tubules and connective tissue were identified by visual inspection also measured with the “lasso” tool.

### Tissue sectioning

Fixed, paraffin-embedded tissue was sectioned on a microtome at a thickness of 10 μm and placed in Eppendorf tubes at +4 °C until RNA extraction. A total of 20–50 μm of tissue was used. Serial sections of each tissue were collected before and after the collection on membranes and stored at −20 °C for subsequent histological assessment of cellularity.

### RNA extraction

Total RNA was extracted using the Recover All Total Nucleic Acid Isolation Kit for FFPE (Thermo Fisher Scientific, MS, USA) according to the manufacturer’s recommendations. RNA was eluted in LoBind tubes using nuclease-free water. To assess the quantity and quality of the samples, the Qubit RNA HS Assay Kit (Thermo Fisher Scientific) and the Agilent RNA 6000 Pico Kit (Agilent Technologies, CA, USA), respectively, were used.

### Library preparation

The Ovation Human FFPE RNA-Seq Multiplex Systems (NuGEN, Leek, Netherlands) were used for library preparation from 150–200 ng of total RNA according to the manufacturer’s recommendations with the following modifications: The cDNA samples were fragmented to yield sizes of approximately 150 bp using the Covaris E210 sonicator (Covaris Ltd., Brighton, UK). For the final library amplification, 20 cycles of amplification were used. An extra DNA purification was performed with a 1.4 times overload of Agencourt AMPure XP beads (Beckman Coulter, IN, USA) to get rid of adapters and primers. Samples were pooled in groups of five with equal amount of each. We performed 150 nt paired-end read sequencing on the Illumina HiSeq 2000 sequencing platform (Illumina, CA, USA).

### Analysis of RNA-sequencing data

Reads were demultiplexed using a custom perl script and adapter sequences trimmed using AdapterRemoval v1.5^41^. Reads were mapped to the human reference genome (GRCh38) in which the pseudoautosomal region (PAR) on the Y-chromosome (PARY; chrY:10001-2781479, chrY:56887903-57217415) were masked using STAR v. 2.5.2b^42^. Read counts were summarized using HTseq v. 0.6.0^43^.

Data were analyzed in the R software v. 3.3.2 (http://www.r-project.org). Briefly, the count data was loaded into the limma/edgeR package^44,45^ and heatmaps were generated with the package gplots^46^ using the heatmap.2 function with the “complete” hierarchical clustering method. Based on this analysis, seven KS samples and two CMC were excluded (Fig. 1a and Supplementary Fig. S1). To avoid accidental findings, we decided to include only transcripts with at least 1 count per million (CPM) in at least two samples in the overexpressing group. This left a total of 22,020 transcripts in the analysis. In Table 1, the library sizes are shown for all samples.

The R package limma^47^ was used to identify DETs and fold changes were calculated as the mean expression values for the two groups of interest and then further log_2_-transformed. A *p*-value < 0.01 and a log_2_ fold change (logFC) ≥ 1.5 or ≤ 1.5 was used as cutoff. Enrichment analyses were performed using mroast and camera functions. XCI status was acquired from ref. ^24^ and GENCODE transcript biotypes were obtained from Ensembl using the biomaRt package in R^48,49^. A hypergeometric test using the phyper function in R was used to test for enrichment of transcript biotypes. Expression of known Sertoli, Leydig, germ cell, and peritubular/blood cell markers in KS, CMC, SCO, and Norm were plotted with GraphPad Prism version 4 (GraphPad Software Inc, CA, USA).

DETs were identified between KS, CMC, SCO, and Norm as performed with the KS vs. CMC, and the overlap was identified in R. The relative expression level of transcripts that overlapped was visualized with the heatmap.2 function using the “manhattan” distance matrix and the “ward.D2” hierarchical clustering method. The overlap was visualized using the interactive venn website (http://www.interactivenn.net/, accessed 2 Sept 2017). Overrepresentation of GO terms^50^ (the “Biological Process” terms) was tested using the DAVID Bioinformatics Resources^51,52^.

Data has been deposited in the GEO public database with the accession number GSE103905.

## Electronic supplementary material

Supplementary Tables

Supplementary Figures
