# Supplementary material for: Transcriptome analysis of the adult human Klinefelter testis and cellularity-matched controls reveals disturbed differentiation of Sertoli- and Leydig cells
Source: Cell Death Dis. 2018 May 22;9(6):586. doi: 10.1038/s41419-018-0671-1 (PMC5964117; doi:10.1038/s41419-018-0671-1)
Supplement: Supplementary file 1 — Supplementary Tables [file 41419_2018_671_MOESM1_ESM.docx]

**Supplementary Table S1: Upregulated transcripts in adult KS versus CMC**

| **Gene symbol** | **Synonym(s)** | **ENSG** | **Location** | **logFC** | **logCPM** | **p-value** | **XCI** | **RNA biotype** |
| --- | --- | --- | --- | --- | --- | --- | --- | --- |
| XIST | LINC00001 | ENSG00000229807 | Xq13.2 | 11.328 | 9.3372 | 1.7E-39 | Mostly S | lincRNA |
| CTD-2144E22.9 | AC135776.4 | ENSG00000279165 | 16p11.2 | 12.064 | 6.0716 | 4.8E-09 |  | TEC |
| FAM9A | TEX39A | ENSG00000183304 | Xp22.31 | 11.477 | 5.4882 | 3.5E-06 | Nd, dis | Protein-coding |
| LINC01239 |  | ENSG00000234840 | 9p21.3 | 10.579 | 4.6026 | 8.0E-06 |  | lincRNA, retained intron |
| GABRA1 | EJM5 | ENSG00000022355 | 5q34 | 10.314 | 4.3423 | 1.2E-05 |  | Protein-coding, processed transcript, retained intron, nonsense-mediated decay |
| FOSB | AP-1, GOSB | ENSG00000125740 | 19q13.32 | 7.2227 | 6.3041 | 5.2E-05 |  | Protein-coding, retained intron |
| POU3F4 | DFN3, OTF9, BRN4 | ENSG00000196767 | Xq21.1 | 9.8380 | 3.8779 | 7.1E-05 | Mostly S | Protein-coding |
| CTD-2619J13.8 | AC012313.3 | ENSG00000268230 | 19q13.43 | 7.6092 | 5.7295 | 9.5E-05 |  | Processed transcript |
| RP11-814E24.1 | AC093392.1 | ENSG00000203462 | 7p11.2 | 7.9726 | 5.2645 | 1.2E-04 |  | Unprocessed pseudogene |
| DACH2 |  | ENSG00000126733 | Xq21.2 | 9.5840 | 3.6324 | 1.5E-04 | Nd, dis | Protein-coding, nonsense-mediated decay |
| RN7SL399P |  | ENSG00000239542 | 17q21.2 | 10.148 | 4.1793 | 1.7E-04 |  | miscRNA |
| LINC00940 |  | ENSG00000235049 | 12p13.33 | 5.0640 | 5.2746 | 1.7E-04 |  | lincRNA |
| RP11-513G11.4 | LINC02036 | ENSG00000225742 | 3q29 | 10.024 | 4.0594 | 2.0E-04 |  | lincRNA |
| SNORA63 |  | ENSG00000252448 | 1p34.3 | 9.5231 | 3.5732 | 2.1E-04 |  | snoRNA |
| RP11-521L9.2 | AC009137.2 | ENSG00000266801 | 16q22.1 | 9.3837 | 3.4393 | 3.0E-04 |  | Sense intronic |
| OR10H5 |  | ENSG00000172519 | 19p13.2 | 9.4940 | 3.5455 | 3.0E-04 |  | Protein-coding |
| RP11-394B2.1 | AC020763.4 | ENSG00000279569 | 16q22.1 | 9.3214 | 3.3794 | 3.7E-04 |  | TEC |
| RP11-338K13.1 | AC097381.3 | ENSG00000273267 | 4p16.1 | 9.9791 | 4.0135 | 3.7E-04 |  | Antisense |
| RNU6-1013P |  | ENSG00000206907 | 9q31.3 | 10.027 | 4.0596 | 4.0E-04 |  | snRNA |
| RP11-636O21.2 | LINC01902 | ENSG00000283503 | 18q12.3 | 5.5384 | 6.0795 | 4.6E-04 |  | lincRNA |
| CTA-280A3.2 | FP325330.3 | ENSG00000279712 | 22q13.31 | 6.9995 | 4.3095 | 4.9E-04 |  | lincRNA |
| C4orf22 |  | ENSG00000197826 | 4q21.21 | 9.0930 | 3.1607 | 6.0E-04 |  | Protein-coding, processed transcript, nonsense-mediated decay |
| RP11-15H7.2 | AL390205.1 | ENSG00000205695 | 6q24.1 | 9.6764 | 3.7205 | 6.2E-04 |  | Processed pseudogene |
| ADAP1 | CENTA1, GCS1L | ENSG00000105963 | 7p22.3 | 4.6747 | 7.1572 | 6.8E-04 |  | Protein-coding, processed transcript, retained intron |
| RP11-391L3.3 | AC092139.4 | ENSG00000279476 | 16q23.2 | 9.0086 | 3.0823 | 8.2E-04 |  | TEC |
| CLCA3P | CLCA3 | ENSG00000153923 | 1p22.3 | 7.1720 | 3.8892 | 8.5E-04 |  | Processed transcript, transcribed unprocessed pseudogene |
| TFAP2D | TFAP2BL1 | ENSG00000008197 | 6p12.3 | 9.5318 | 3.5824 | 8.8E-04 |  | Protein-coding, processed transcript |
| P4HA2 |  | ENSG00000072682 | 5q31.1 | 4.4687 | 5.5775 | 9.0E-04 |  | Protein-coding, processed transcript, retained intron |
| JSRP1 | JP-45 | ENSG00000167476 | 19p13.3 | 9.5327 | 3.5817 | 9.3E-04 |  | Protein-coding, processed transcript |
| RP11-461F11.2 | AC026523.2 | ENSG00000279091 | 15q26.2 | 5.3516 | 5.4510 | 9.4E-04 |  | TEC |
| SGO1-AS1 | SGOL1-AS1 | ENSG00000231304 | 3p24.3 | 9.4426 | 3.5014 | 9.8E-04 |  | lincRNA, antisense |
| IGF2 | IGF-II, C11orf43 | ENSG00000167244 | 11p15.5 | 2.1263 | 12.390 | 0.0010 |  | Protein-coding |
| EGR3 | PILOT | ENSG00000179388 | 8p21.3 | 4.0717 | 4.9924 | 0.0010 |  | Protein-coding, processed transcript |
| HOXB13 |  | ENSG00000159184 | 17q21.32 | 8.0813 | 4.3197 | 0.0011 |  | Protein-coding |
| RP11-38O23.4 | AC244636.2 | ENSG00000233139 | Xp11.23 | 5.9958 | 6.4321 | 0.0011 | Nd, dis | Processed pseudogene |
| SI |  | ENSG00000090402 | 3q26.1 | 8.4148 | 3.9873 | 0.0011 |  | Protein-coding, retained intron, nonsense-mediated decay |
| GPR182 | AM-R, ADMR | ENSG00000166856 | 12q13.3 | 9.4345 | 3.4857 | 0.0013 |  | Protein-coding |
| RP11-340L3.1 | AL049781.1 | ENSG00000280281 | 14q12 | 7.3763 | 6.0107 | 0.0013 |  | TEC |
| CHRM2 |  | ENSG00000181072 | 7q33 | 5.5569 | 4.3381 | 0.0014 |  | Protein-coding, processed transcript |
| ACHE | YT | ENSG00000087085 | 7q22.1 | 8.5777 | 2.6768 | 0.0017 |  | Protein-coding, processed transcript, nonsense-mediated decay |
| FUT9 |  | ENSG00000172461 | 6q16.1 | 9.1924 | 3.2623 | 0.0018 |  | Protein-coding, processed transcript |
| TEKT1 |  | ENSG00000167858 | 17qp13.1 | 9.3202 | 3.3750 | 0.0019 |  | Protein-coding, retained intron, nonsense-mediated decay |
| RP11-297H3.3 | LINC01681 | ENSG00000233985 | 1q24.2 | 9.2772 | 3.3342 | 0.0019 |  | lincRNA |
| RNA5SP219 |  | ENSG00000212242 | 6q23.3 | 9.2339 | 3.2930 | 0.0020 |  | rRNA |
| WDR87 | NYD-SP11 | ENSG00000171804 | 19q13.3 | 5.4170 | 5.1344 | 0.0020 |  | Protein-coding, retained intron |
| ASTN1 | ASTN | ENSG00000152092 | 1q25.2 | 5.0757 | 4.4746 | 0.0021 |  | Protein-coding, processed transcript |
| KYNU |  | ENSG00000115919 | 2q22.2 | 4.5081 | 4.5450 | 0.0022 |  | Protein-coding, retained intron, nonsense-mediated decay |
| HMGB1P33 |  | ENSG00000261174 | 15q21.3 | 9.2675 | 3.3241 | 0.0022 |  | Processed pseudogene |
| RP11-214O1.3 | AC005224.3 | ENSG00000266378 | 17p12 | 6.7047 | 5.5170 | 0.0022 |  | lincRNA |
| B3GALT5 | GLCT5, B3GalT‑V, B3T5 | ENSG00000183778 | 21q22.2 | 4.7361 | 4.8727 | 0.0023 |  | Protein-coding, processed transcript |
| BNIP3P36 |  | ENSG00000271661 | 19p12 | 9.1462 | 3.2098 | 0.023 |  | Processed pseudogene |
| JUNB |  | ENSG00000171223 | 19p13.13 | 3.0146 | 5.9054 | 0.0023 |  | Protein-coding |
| RPL3P9 |  | ENSG00000240540 | 8q21.13 | 7.3482 | 3.6065 | 0.0023 |  | Processed pseudogene |
| RP4-781K5.7 | AL160408.6 | ENSG00000282097 | 1q42.3 | 9.1770 | 3.2378 | 0.0024 |  | lincRNA |
| NSUN7 | FLJ14001 | ENSG00000179299 | 4p14 | 9.0655 | 3.1416 | 0.0024 |  | Protein-coding, processed transcript, retained intron |
| GALR1 | GALNR1, GALNR | ENSG00000166573 | 18q23 | 9.1060 | 3.1795 | 0.0025 |  | Protein-coding, processed transcript |
| KDM4A-AS1 |  | ENSG00000236200 | 1p34.2-p34.1 | 9.1741 | 3.2346 | 0.0026 |  | Antisense |
| LINC00599 | Rncr3 | ENSG00000253230 | 8p23.1 | 8.6860 | 2.7728 | 0.0027 |  | lincRNA |
| RP11-489E7.1 | AC131254.2 | ENSG00000253604 | 8p12 | 5.9228 | 4.3190 | 0.0027 |  | lincRNA |
| LINC00562 |  | ENSG00000260388 | 13q14.2 | 6.5338 | 5.0014 | 0.0027 |  | lincRNA |
| OR6B1 | OR7-3 | ENSG00000221813 | 7q35 | 9.0242 | 3.0953 | 0.0028 |  | Protein-coding |
| QRFPR | GPR103 | ENSG00000186867 | 4q27 | 9.1491 | 3.2104 | 0.0028 |  | Protein-coding, retained intron, nonsense-mediated decay |
| RRH | peropsin | ENSG00000180245 | 4q25 | 9.0106 | 3.0806 | 0.0030 |  | Protein-coding |
| HHLA1 | PLA2L | ENSG00000132297 | 8q24.22 | 8.9794 | 3.0514 | 0.0030 |  | Protein-coding, retained intron |
| CRYBB2 | CRYB2A, CCA2 | ENSG00000244752 | 22q11.23 | 7.6623 | 3.8958 | 0.0030 |  | Protein-coding |
| CCDC38 | FLJ40089 | ENSG00000165972 | 12q23.1 | 7.0457 | 3.3184 | 0.0031 |  | Protein-coding, processed transcript, retained intron, nonsense-mediated decay |
| OLFM3 | NOE3 | ENSG00000118733 | 1p21.1 | 8.9945 | 3.0647 | 0.0032 |  | Protein-coding, processed transcript |
| RP11-461L13.4 | AC080078.1 | ENSG00000270302 | 4q31.3 | 9.0159 | 3.0841 | 0.0032 |  | lincRNA |
| RP11-505K1.1 | AL360085.1 | ENSG00000271498 | 6q21 | 9.0537 | 3.1196 | 0.0032 |  | Processed pseudogene |
| RP11-382E9.1 | AC099673.1 | ENSG00000231718 | 1q32.1 | 9.1618 | 3.2221 | 0.0032 |  | lincRNA |
| CTC-304I17.6 | AC024610.2 | ENSG00000279668 | 17q12 | 7.4087 | 3.2525 | 0.0034 |  | lincRNA |
| AC007386.2 | LINC02245 | ENSG00000237638 | 2p14 | 5.7166 | 5.0499 | 0.0036 |  | lincRNA |
| ZNF385D | ZNF659, FLJ22419 | ENSG00000151789 | 3p24.3 | 3.5760 | 5.5527 | 0.0037 |  | Protein-coding, processed transcript, nonsense-mediated decay |
| RP3-323A16.1 | Z99755.3 | ENSG00000279184 | 22q12.3 | 3.6792 | 5.0354 | 0.0037 |  | Antisense |
| SPATA16 | NYD-SP12 | ENSG00000144962 | 3q26.31 | 8.7787 | 2.8721 | 0.0039 |  | Protein-coding |
| OTX2-AS1 | OTX2OS1 | ENSG00000248550 | 14q22.3 | 7.1780 | 4.1762 | 0.0039 |  | lincRNA |
| KCNMB2 |  | ENSG00000197584 | 3q26.32 | 6.0025 | 3.8849 | 0.0040 |  | Protein-coding, processed transcript, nonsense-mediated decay |
| CLDN4 | WBSCR8, CPE‑R, CPETR1 | ENSG00000189143 | 7q11.23 | 5.8409 | 3.6676 | 0.0041 |  | Protein-coding, processed transcript |
| CTD-2161F6.2 | AC026719.1 | ENSG00000248311 | 5p15.32 | 8.8209 | 2.9019 | 0.0041 |  | lincRNA |
| AC079776.3 | LINC01856 | ENSG00000237574 | 2q21.1 | 4.1504 | 4.6517 | 0.0041 |  | lincRNA |
| RP11-13A1.1 | LINC02458 | ENSG00000246363 | 12q21.33 | 8.9207 | 2.9929 | 0.0042 |  | lincRNA |
| LINC01554 | FIS, C5orf27 | ENSG00000236882 | 5q15 | 7.1021 | 3.3964 | 0.0042 |  | lincRNA |
| MED15P8 |  | ENSG00000248796 | 2q21.1 | 8.8103 | 2.8905 | 0.0043 |  | Unprocessed pseudogene |
| C10orf53 |  | ENSG00000178645 | 10q11.23 | 8.3315 | 2.4574 | 0.0044 |  | Protein-coding |
| PNMA5 | KIAA1934 | ENSG00000198883 | Xq28 | 8.7831 | 2.8653 | 0.0044 | Mostly S | Protein-coding |
| PSCA |  | ENSG00000167653 | 8q24.3 | 8.8820 | 2.9561 | 0.0045 |  | Protein-coding, processed transcript |
| CTB-113D17.1 | AC005162.3 | ENSG00000272568 | 7p14.3 | 8.7978 | 2.8888 | 0.0045 |  | Antisense |
| PLA2G4F |  | ENSG00000168907 | 15q15.1 | 8.8374 | 2.9258 | 0.0046 |  | Protein-coding, retained intron, nonsense-mediated decay |
| GPR158 | KIAA1136 | ENSG00000151025 | 10p12.1 | 4.9487 | 4.0191 | 0.0046 |  | Protein-coding, processed transcript |
| CTC-339D2.1 | LINC02039 | ENSG00000248107 | 5q23.2 | 8.7436 | 2.8384 | 0.0046 |  | lincRNA |
| SLC4A4 | SLC4A5, NBC2, HNBC1, NBC1 | ENSG00000080493 | 4q13.3 | 7.8013 | 3.3797 | 0.0048 |  | Protein-coding, processed transcript |
| FRMD7 | NYS, FLJ43346 | ENSG00000165694 | Xq26.2 | 8.7377 | 2.8327 | 0.0048 | VE | Protein-coding |
| MEIOC | C17orf104, FLJ35848 | ENSG00000180336 | 17q21.31 | 8.7410 | 2.8240 | 0.0049 |  | Protein-coding, processed transcript, nonsense-mediated decay |
| DPP4 | DPPIV, CD26, ADCP2 | ENSG00000197635 | 2q24.2 | 6.2512 | 3.4135 | 0.0049 |  | Protein-coding, processed transcript, retained intron, nonsense-mediated decay |
| RP11-194N12.2 | AC107993.1 | ENSG00000267222 | 17q21.2 | 8.7916 | 2.8707 | 0.0050 |  | Sense intronic |
| RALYL | HNRPCL3 | ENSG00000184672 | 8q21.2 | 5.8944 | 3.9222 | 0.0050 |  | Protein-coding, retained intron |
| APLN | XNPEP2 | ENSG00000171388 | Xq26.1 | 3.8452 | 5.2591 | 0.0050 | Mostly S | Protein-coding |
| RP11-407B7.1 | LINC02028 | ENSG00000230102 | 3q29 | 6.4343 | 3.5391 | 0.0050 |  | lincRNA, retained intron |
| CTC-435M10.10 | AC011462.3 | ENSG00000268987 | 19q13.2 | 6.4968 | 4.4898 | 0.0050 |  | Sense intronic |
| SHOX2 | OG12X, SHOT | ENSG00000168779 | 3q25.32 | 8.0889 | 2.2369 | 0.0051 |  | Protein-coding, retained intron |
| RP11-327P2.5 | AL162377.1 | ENSG00000231856 | 13q14.3 | 5.8099 | 3.3324 | 0.0052 |  | Antisense |
| MT-TY | MTTY | ENSG00000210144 | MT: 5,826-5,891 | 1.7596 | 8.2451 | 0.0052 |  | Mt-tRNA |
| RP11-366L20.2 | AC090673.1 | ENSG00000197301 | 12q14.3 | 7.9341 | 2.0842 | 0.0054 |  | Antisense |
| THAP12P5 | PRKRIRP5 | ENSG00000218020 | 6p24.2 | 8.8346 | 2.9104 | 0.0055 |  | Transcribed processed pseudogene |
| DLK1 | Delta1, FA1 | ENSG00000185559 | 14q32.2 | 1.5920 | 10.2932 | 0.0056 |  | Protein-coding, retained intron |
| RP11-445O3.3 | AC106799.3 | ENSG00000260763 | 5p15.32 | 7.7073 | 3.2823 | 0.0058 |  | lincRNA |
| TDRD9 | FLJ36164, C14orf75, NET54 | ENSG00000156414 | 14q32.33 | 6.1334 | 3.4433 | 0.0059 |  | Protein-coding, processed transcript, retained intron |
| RPSAP71 |  | ENSG00000254256 | 5q35.1 | 8.1442 | 2.2862 | 0.0060 |  | Processed pseudogene |
| LINC01579 |  | ENSG00000258754 | 15q26.2-q26.1 | 5.2470 | 4.1125 | 0.0061 |  | lincRNA |
| CYLC2 |  | ENSG00000155833 | 9q31.1 | 8.5932 | 2.6857 | 0.0062 |  | Protein-coding, processed transcript |
| RP11-44K6.3 | AC007991.3 | ENSG00000253939 | 8p11.21 | 6.6597 | 4.4943 | 0.0063 |  | Antisense |
| CGA | FSHA, HCG, LHA, GPHA1 | ENSG00000135346 | 6q14.3 | 8.5158 | 2.6272 | 0.0063 |  | Protein-coding, retained intron |
| PCDH15 | DFNB23, CDHR15, USH1F | ENSG00000150275 | 10q21.1 | 5.7368 | 3.8355 | 0.0067 |  | Protein-coding, processed transcript, retained intron, nonsense-mediated decay |
| CTD-3116E22.7 | AC008759.2 | ENSG00000267612 | 19p13.2 | 8.5028 | 2.6145 | 0.0068 |  | lincRNA |
| HELLPAR | LINC-HELLP | ENSG00000281344 | 12q32.2 | 1.8416 | 7.0023 | 0.0068 |  | Macro lncRNA |
| RP11-399E6.4 | AC093151.2 | ENSG00000229901 | 1p34.2 | 7.5562 | 3.1430 | 0.0070 |  | Antisense |
| LBX1 | HPX6, LBX1H | ENSG00000138136 | 10q24.32 | 5.2345 | 3.9811 | 0.0070 |  | Protein-coding |
| RP11-307C19.1 | AC046168.1 | ENSG00000259362 | 15q24.3 | 5.9008 | 4.1691 | 0.0070 |  | lincRNA |
| SAGE4P |  | ENSG00000224539 | Xq26.3 | 8.3796 | 2.5029 | 0.0072 | Nd, dis | Unprocessed pseudogene |
| CYP3A4 | CYP3A3 | ENSG00000160868 | 7q22.1 | 8.5125 | 2.6094 | 0.0072 |  | Protein-coding, retained intron |
| LINC00649 |  | ENSG00000237945 | 21q22.11 | 3.2739 | 5.0737 | 0.0074 |  | Antisense |
| AC009299.3 | LINC01806 | ENSG00000227403 | 2q24.2 | 8.3835 | 2.5055 | 0.0075 |  | lincRNA |
| RP1-302D9.1 | Z82198.1 | ENSG00000228622 | 22q12.3 | 8.5423 | 2.6356 | 0.0078 |  | lincRNA |
| CHST8 | GALNAC-4-ST1 | ENSG00000124302 | 19q13.11 | 8.3997 | 2.5063 | 0.0078 |  | Protein-coding, processed transcript |
| NEFL | PPP1R110, CMT1F, NF68 | ENSG00000277586 | 8p21.2 | 8.2614 | 2.3979 | 0.0079 |  | Protein-coding, retained intron |
| AC010148.1 |  | ENSG00000235726 | 2q37.2 | 5.6294 | 3.8368 | 0.0081 |  | lincRNA, retained intron |
| CHEK1 | CHK1 | ENSG00000149554 | 11q24.2 | 5.1114 | 4.1281 | 0.0081 |  | Protein-coding, retained intron, nonsense-mediated decay |
| CTB-35F21.3 | AC008667.3 | ENSG00000251387 | 5q31.2 | 8.4664 | 2.5654 | 0.0081 |  | lincRNA |
| PRKG2 | PRKGR2 | ENSG00000138669 | 4q21.21 | 8.2698 | 2.4033 | 0.0081 |  | Protein-coding, processed transcript |
| RP11-83N9.5 | AL138781.1 | ENSG00000260193 | 9q34.3 | 8.2926 | 2.4229 | 0.0082 |  | lincRNA |
| LINC01299 |  | ENSG00000254081 | 8q13.1 | 8.3700 | 2.4922 | 0.0083 |  | lincRNA |
| RP11-415I12.2 | AC084357.3 | ENSG00000255583 | 12q14.2 | 8.2934 | 2.4234 | 0.0083 |  | Processed transcript, transcribed unprocessed pseudogene |
| FBXO43 | Fbx43 | ENSG00000156509 | 8q22.2 | 8.2552 | 2.3889 | 0.0086 |  | Protein-coding, retained intron, nonsense-mediated decay |
| AIFM3 | AIFL, FLJ30473 | ENSG00000183773 | 22q11.21 | 8.3613 | 2.4686 | 0.0089 |  | Protein-coding, processed transcript, retained intron, nonsense-mediated decay |
| XKR4 | KIAA1889 | ENSG00000206579 | 8q12.1 | 3.4360 | 5.0028 | 0.0091 |  | Protein-coding, processed transcript |
| EHD4-AS1 |  | ENSG00000259883 | 15q15.1 | 8.2790 | 2.4093 | 0.0091 |  | Antisense |
| KLRD1 | CD94 | ENSG00000134539 | 12p13.2 | 4.4652 | 4.1806 | 0.0091 |  | Protein-coding, processed transcript, retained intron, nonsense-mediated decay |
| OR2AT4 |  | ENSG00000171561 | 11q13.4 | 8.1878 | 2.3278 | 0.0091 |  | Protein-coding |
| MS4A8 | CD20L5, MS4A8B, MS4A4 | ENSG00000166959 | 11q12.2 | 8.1546 | 2.2987 | 0.0092 |  | Protein-coding, retained intron, nonsense-mediated decay |
| FEZF1-AS1 |  | ENSG00000230316 | 7q31.32 | 7.5307 | 1.7433 | 0.0093 |  | Antisense |
| RAD21L1 | RAD21L | ENSG00000244588 | 20p13 | 8.1905 | 2.3295 | 0.0094 |  | Protein-coding, processed transcript |
| LRRIQ1 | KIAA1801 | ENSG00000133640 | 12q21.31 | 8.2857 | 2.3998 | 0.0095 |  | Protein-coding, processed transcript, retained intron |
| PLEKHS1 | C10orf81 | ENSG00000148735 | 10q25.3 | 8.3237 | 2.4464 | 0.0095 |  | Protein-coding |
| RP11-454H19.2 | AC080100.1 | ENSG00000279675 | 11p12 | 8.1360 | 2.2814 | 0.0095 |  | TEC |
| STIL | MCPH7, SIL | ENSG00000123473 | 1p33 | 3.4362 | 4.6331 | 0.0096 |  | Protein-coding, retained intron |
| RPS3AP23 |  | ENSG00000218351 | 6q24.1 | 6.8630 | 1.1846 | 0.0096 |  | Processed transcript, transcribed processed pseudogene |
| TFAP2A | AP2TF, AP-2, TFAP2 | ENSG00000137203 | 6p24.3 | 8.2555 | 2.3728 | 0.0096 |  | Protein-coding, processed transcript, retained intron, nonsense-mediated decay |
| BORCS8 | MEF2BNB | ENSG00000254901 | 19p13.11 | 2.5883 | 5.5038 | 0.0096 |  | Protein-coding, processed transcript, retained intron, nonsense-mediated decay |
| RP11-430C1.1 | LINC02141 | ENSG00000261807 | 16q21 | 8.2220 | 2.3575 | 0.0097 |  | lincRNA |
| MTND5P21 |  | ENSG00000255123 | 11p15.4 | 8.1819 | 2.3213 | 0.0097 |  | Processed pseudogene |
| RP11-632K21.6 | AC069304.2 | ENSG00000270990 | 7q35.1 | 8.1922 | 2.3306 | 0.0097 |  | Processed pseudogene |
| KCNJ6 | GIRK2, KATP2, KCNJ7, BIR1 | ENSG00000157542 | 21q22.13 | 3.1056 | 4.9590 | 0.0099 |  | Protein-coding |
| RP11-89M22.3 | AC090109.1 | ENSG00000258115 | 12q21.1 | 8.1289 | 2.2741 | 0.0100 |  | Antisense |

XCI: X‑chromosome inactivation status (25); Nd, dis: no or discordant data; S: silenced; VE: variable escape.

**Supplementary Table S2: Downregulated transcripts in adult KS versus CMC**

| **Gene symbol** | **Synonym(s)** | **ENSG** | **Location** | **logFC** | **logCPM** | **p-value** | **XCI** | **RNA biotype** |
| --- | --- | --- | --- | --- | --- | --- | --- | --- |
| IGHG3 |  | ENSG00000211897 | 14q32.33 | -10.275 | 6.1706 | 5.8E-06 |  | IG C genes |
| GLI1 | GLI | ENSG00000111087 | 12q13.3 | -9.4914 | 3.5506 | 2.0E-04 |  | Protein-coding, retained intron |
| IGKC | HCAK1 | ENSG00000211592 | 2p11.2 | -8.7366 | 10.191 | 4.2E-04 |  | IG C genes |
| RP11-235E17.4 | AC027796.1 | ENSG00000261916 | 17p13.2 | -8.9713 | 3.0566 | 9.5E-04 |  | Sense intronic |
| C3 | CPAMD1, C3b, C3a, ARMD9 | ENSG00000125730 | 19p13.3 | -2.5901 | 6.4424 | 9.5E-04 |  | Protein-coding, processed transcript, retained intron |
| ITLN1 | HL-1, ITLN, LFR | ENSG00000179914 | 1q23.3 | -8.9695 | 3.0600 | 0.0010 |  | Protein-coding, processed transcript |
| C18orf32 | FLJ23458 | ENSG00000177576 | 18q21.2 | -8.7497 | 2.8516 | 0.0014 |  | Protein-coding |
| MYH11 | SMHC, SMMHC | ENSG00000133392 | 16p13.11 | -1.5446 | 8.4376 | 0.0015 |  | Protein-coding, processed transcript, retained intron |
| RP11-809C18.3 | AL358216.1 | ENSG00000225140 | 10p15.3 | -8.7296 | 2.8283 | 0.0019 |  | Antisense |
| MTG1 | GTPBP7 | ENSG00000148824 | 10q26.3 | -8.7502 | 2.8455 | 0.0020 |  | Protein-coding, retained intron |
| AC093818.1 |  | ENSG00000225205 | 2q31.1 | -8.4955 | 2.6168 | 0.0025 |  | Antisense |
| ASMTL-AS1 | CXYorf2 | ENSG00000236017 | Xp22.33 | -8.3555 | 2.4907 | 0.0031 | PAR1 | Antisense |
| BX322557.10 |  | ENSG00000215447 | 21q22.3 | -8.3240 | 2.4572 | 0.0034 |  | lincRNA, antisense |
| DUSP19 | DUSP17, SKRP1 | ENSG00000162999 | 2q32.1 | -8.2705 | 2.4128 | 0.0036 |  | Protein-coding, processed transcript |
| RDH5 | SDR9C5, RDH1, HSD17B9 | ENSG00000135437 | 12q13.2 | -8.2859 | 2.4251 | 0.0038 |  | Protein-coding, processed transcript, retained intron, nonsense-mediated decay |
| TOE1 |  | ENSG00000132773 | 1p34.1 | -8.2772 | 2.4168 | 0.0038 |  | Protein-coding, processed transcript |
| LINC00884 |  | ENSG00000233058 | 3q29 | -8.2544 | 2.3932 | 0.0040 |  | Antisense |
| CLEC4GP1 |  | ENSG00000268297 | 19p13.2 | -8.9440 | 3.0382 | 0.0040 |  | Unprocessed pseudogene |
| AF129075.5 |  | ENSG00000231125 | 21q21.3 | -8.2382 | 2.3784 | 0.0042 |  | Sense intronic |
| RP11-43D4.3 | AC079316.2 | ENSG00000279176 | 12q23.3 | -8.1823 | 2.3371 | 0.0043 |  | TEC |
| NFAM1 | CNAIP | ENSG00000235568 | 22q13.2 | -8.4144 | 2.5333 | 0.0043 |  | Protein-coding, nonsense-mediated decay |
| RP11-1113L8.1 | AC025627.1 | ENSG00000262769 | 17p11.2 | -8.3396 | 2.4710 | 0.0043 |  | Antisense |
| RP11-254F19.4 | AC025280.2 | ENSG00000279622 | 16q24.1 | -8.8379 | 2.9407 | 0.0044 |  | TEC |
| TRMT61B | FLJ20628 | ENSG00000171103 | 2p23.2 | -8.1173 | 2.2729 | 0.0047 |  | Protein-coding, processed transcript, retained intron, nonsense-mediated decay |
| UBE2S | E2-EPF | ENSG00000108106 | 19q13.42 | -8.2485 | 2.3954 | 0.0049 |  | Protein-coding, processed transcript |
| AC008781.7 |  | ENSG00000228737 | 5q31.3 | -8.3568 | 2.4786 | 0.0051 |  | Antisense |
| KCTD21-AS1 |  | ENSG00000246174 | 11q14.1 | -8.2709 | 2.4049 | 0.0051 |  | Antisense |
| RAB11FIP1P1 |  | ENSG00000228492 | Xq13.2 | -8.3099 | 2.4396 | 0.0054 | Nd, dis | Processed pseudogene |
| ALDOB |  | ENSG00000136872 | 9q31.1 | -8.0199 | 2.1818 | 0.0055 |  | Protein-coding, processed transcript |
| CELF2-AS1 | C10orf31 | ENSG00000181800 | 10p14 | -8.1272 | 2.2726 | 0.0059 |  | Antisense |
| RPL23AP64 |  | ENSG00000240970 | 11q23.3 | -7.9080 | 2.0883 | 0.0059 |  | Processed pseudogene |
| IGLV3-1 |  | ENSG00000211673 | 22q11.22 | -8.7345 | 2.8387 | 0.0059 |  | IG_V gene |
| UBE2CP1 |  | ENSG00000258648 | 14q12 | -8.0288 | 2.1931 | 0.0060 |  | Processed pseudogene |
| RP11-297D21.4 | AC009061.2 | ENSG00000270049 | 16q22.1 | -7.9934 | 2.1583 | 0.0060 |  | Antisense |
| RP1-67K17.4 | AL023584.2 | ENSG00000237851 | 6q24.2 | -8.0907 | 2.2438 | 0.0062 |  | lincRNA |
| SNORA47 | HBI-115 | ENSG00000238961 | 5q13.3 | -2.5176 | 6.6178 | 0.0063 |  | snoRNA |
| PRB4 |  | ENSG00000230657 | 12p13.2 | -7.8755 | 2.0545 | 0.0064 |  | Protein-coding |
| SDR39U1 | HCDI, C14orf124 | ENSG00000100445 | 14q12 | -7.9726 | 2.1358 | 0.0065 |  | Protein-coding, processed transcript, retained intron |
| RP11-503N18.5 | AL645924.2 | ENSG00000251229 | 4p16.3 | -7.8653 | 2.0476 | 0.0067 |  | Processed pseudogene |
| ZFPM2-AS1 |  | ENSG00000251003 | 8q23.1 | -7.9100 | 2.0813 | 0.0067 |  | Antisense, retained intron |
| BRICD5 | C16orf79 | ENSG00000182685 | 16p13.3 | -7.9905 | 2.1605 | 0.0068 |  | Protein-coding, retained intron |
| PRH2 |  | ENSG00000134551 | 12p13.2 | -7.9496 | 2.1182 | 0.0069 |  | Protein-coding |
| CTD-2265D6.2 | AC093225.1 | ENSG00000250860 | 5p13.1 | -7.8119 | 1.9980 | 0.0069 |  | Processed pseudogene |
| AP000688.14 |  | ENSG00000230212 | 21q22.12 | -7.8971 | 2.0734 | 0.0069 |  | Sense intronic |
| ZBTB32 | TZFP, ZNF538, Rog, FAZF, FAXF | ENSG00000011590 | 19q13.12 | -7.9933 | 2.1515 | 0.0071 |  | Protein-coding, processed transcript, nonsense-mediated decay |
| CTB-92J24.3 | AC011503.1 | ENSG00000269289 | 19p12 | -8.1341 | 2.2832 | 0.0072 |  | Antisense |
| RP11-744O11.2 | AC119744.1 | ENSG00000236241 | 3q26.31 | -7.8559 | 2.0356 | 0.0073 |  | Processed pseudogene |
| MIR1206 | MIRN1206 | ENSG00000283200 | 8q24.21 | -7.9666 | 2.1283 | 0.0073 |  | miRNA |
| CTD-3145H4.1 | AC110053.1 | ENSG00000254076 | 8q24.23 | -7.9213 | 2.0883 | 0.0073 |  | Processed pseudogene |
| KRT8P12 | KRT8L2 | ENSG00000229320 | 3q25.33 | -7.7530 | 1.9515 | 0.0076 |  | Processed transcript, transcribed processed pseudogene |
| KRT18P31 |  | ENSG00000249850 | 5q13.2 | -7.7776 | 1.9647 | 0.0077 |  | Processed pseudogene |
| JAK3 | JAKL, LJAK | ENSG00000105639 | 19p13.11 | -2.6774 | 5.4613 | 0.0077 |  | Protein-coding, processed transcript, retained intron |
| CTD-2522E6.4 | AL513366.1 | ENSG00000237351 | Xp11.3 | -7.7650 | 1.9540 | 0.0077 |  | Processed pseudogene |
| GPR17 |  | ENSG00000144230 | 2q14.3 | -7.7411 | 1.9336 | 0.0079 |  | Protein-coding, processed transcript |
| RP11-85I17.2 | AC021945.1 | ENSG00000279347 | 8q24.12 | -7.8141 | 1.9947 | 0.0080 |  | TEC |
| SLC14A2-AS1 |  | ENSG00000267097 | 18q12.3 | -7.7434 | 1.9352 | 0.0082 |  | Antisense |
| GNAO1 | G-ALPHA-o | ENSG00000087258 | 16q13 | -2.0491 | 6.0433 | 0.0082 |  | Protein-coding, processed transcript, retained intron, nonsense-mediated decay |
| ACTL10 | C20orf134 | ENSG00000182584 | 20q11.22 | -7.6211 | 1.8331 | 0.0083 |  | Protein-coding |
| ITLN2 | HL-2 | ENSG00000158764 | 1q23.3 | -7.7361 | 1.9292 | 0.0083 |  | Protein-coding, processed transcript |
| RP11-379C10.4 | AL360268.1 | ENSG00000230536 | 9q34.11 | -7.8302 | 2.0064 | 0.0084 |  | Antisense |
| DCDC2B |  | ENSG00000222046 | 1p35.2 | -7.6133 | 1.8255 | 0.0084 |  | Protein-coding, retained intron |
| PKD1L3 |  | ENSG00000277481 | 16q22.2 | -7.5817 | 1.8002 | 0.0085 |  | Protein-coding |
| TBC1D27 |  | ENSG00000128438 | 17p11.2 | -7.7004 | 1.8959 | 0.0086 |  | Processed transcript, transcribed unprocessed pseudogene |
| NHS-AS1 |  | ENSG00000230020 | Xp22.13 | -7.5400 | 1.7705 | 0.0087 |  | Antisense |
| RP11-15E18.1 | AC011921.1 | ENSG00000259349 | 17q23.2 | -7.7164 | 1.9091 | 0.0088 |  | Antisense |
| ABCA2 | ABC2 | ENSG00000107331 | 9q34.3 | -2.7076 | 5.6704 | 0.0088 |  | Protein-coding, processed transcript, retained intron, nonsense-mediated decay |
| AC026904.1 | AC026904.1 | ENSG00000233858 | 8q11.21 | -7.6789 | 1.8780 | 0.0089 |  | lincRNA |
| PRKX-AS1 |  | ENSG00000236188 | Xp22.33 | -7.7514 | 1.9417 | 0.0090 | Nd, dis | Antisense |
| AC009120.4 |  | ENSG00000261404 | 16q23.1-q22.3 | -6.6944 | 2.6364 | 0.0090 |  | lincRNA |
| TH | DYT5b | ENSG00000180176 | 11p15.5 | -7.7147 | 1.9080 | 0.0091 |  | Protein-coding, retained intron, nonsense-mediated decay |
| RP11-10E18.7 | AL138955.1 | ENSG00000276809 | 13q32.1 | -7.5633 | 1.7805 | 0.0092 |  | Sense intronic |
| CLRN1-AS1 | UCRP | ENSG00000239265 | 3q25.1 | -7.5143 | 1.7428 | 0.0092 |  | Antisense |
| RP4-613B23.1 | AC006059.1 | ENSG00000230084 | 3p22.1 | -7.7132 | 1.9058 | 0.0095 |  | Antisense |
| RP11-881L2.1 | AP001381.1 | ENSG00000266541 | 18p11.22 | -7.5691 | 1.7843 | 0.0096 |  | Antisense |
| CMB9-22P13.1 | AP000769.1 | ENSG00000173727 | 11q13.1 | -7.4085 | 1.6592 | 0.0096 |  | Processed transcript, transcribed unprocessed pseudogene |
| RP11-1007J8.1 | AC093367.1 | ENSG00000254131 | 8p11.21 | -7.7192 | 1.9097 | 0.0097 |  | Processed pseudogene |
| LONRF3 | RNF127, FLJ22612 | ENSG00000175556 | Xq24 | -7.6579 | 1.8794 | 0.0097 | Mostly S | Protein-coding, processed transcript, nonsense-mediated decay |
| CTD-3035K23.3 | AC080038.3 | ENSG00000279713 | 17q23.2 | -7.5543 | 1.7751 | 0.0098 |  | TEC |
| TOR2A | FLJ14771, TORP1 | ENSG00000160404 | 9q34.11 | -7.6010 | 1.8132 | 0.0098 |  | Protein-coding, retained intron, nonsense-mediated decay |
| AC009950.2 |  | ENSG00000225963 | 2q37.1 | -7.4902 | 1.7191 | 0.0099 |  | Antisense |
| RNF212B | C14orf164 | ENSG00000215277 | 14q11.2 | -7.3837 | 1.6301 | 0.0099 |  | Protein-coding, processed transcript |
| CTA-292E10.8 | Z93930.3 | ENSG00000272858 | 22q12.1 | -7.6058 | 1.8116 | 0.0099 |  | Sense intronic |

XCI: X‑chromosome inactivation status (25); Nd, dis: no or discordant data; PAR1: pseudoautosomal region 1; S: silenced.

**Supplementary Table S3: DETs overlapping with previous studies**

| **Gene symbol** | **ENSG** | **Location** | **Blood** | **D’Aurora 2015 (23)** | **D’Aurora 2017 (24)** | **KS vs. CMC** | | **RNA biotype** |
| --- | --- | --- | --- | --- | --- | --- | --- | --- |
|  |  |  | **Direction** | **Direction** | **Direction** | **logFC** | **p-value** |  |
| XIST | ENSG00000229807 | Xq13.2 | Up^a^ |  |  | 11.3 | 1.7E-39 | lincRNA |
| ITLN1 | ENSG00000179914 | 1q23.3 | Up^b^ |  |  | -9.0 | 0.0010 | Protein coding, processed transcript |
| P4HA2 | ENSG00000072682 | 5q31.1 | Up^c^ |  |  | 4.5 | 0.00090 | Protein coding, processed transcript |
| FBXO43 | ENSG00000156509 | 8q22.2 |  | Up |  | 8.3 | 0.0086 | Protein coding, retained intron, nonsense-mediated decay |
| OR6B1 | ENSG00000221813 | 7q35 |  | Up |  | 9.0 | 0.0028 | Protein coding |
| FOSB | ENSG00000125740 | 19q13.32 |  |  | Up | 7.2 | 5.2E-05 | Protein coding, retained intron |
| FUT9 | ENSG00000172461 | 6q16.1 |  |  | Up | 9.2 | 0.0018 | Protein coding, processed transcript |
| DLK1 | ENSG00000185559 | 14q32.2 |  |  | Up | 1.6 | 0.0056 | Protein coding, retained intron |
| FAM9A | ENSG00000183304 | Xp22.31 |  |  | Down | 11.5 | 3.5E-06 | Protein coding |
| C4orf22 | ENSG00000197826 | 4q21.21 |  |  | Down | 9.1 | 0.00060 | Protein coding, processed transcript, nonsense-mediated decay |
| XKR4 | ENSG00000206579 | 8q12.1 |  |  | Down | 3.4 | 0.0091 | Protein coding, processed transcript |
| PCDH15 | ENSG00000150275 | 10q21.1 |  |  | Down | 5.7 | 0.0067 | Protein coding, processed transcript, retained intron, nonsense-mediated decay |

^a^ Upregulated in Belling *et al*. (14), Huang *et al*. (15), Zitzmann *et al*. (16), Vawter *et al*. (17); ^b^ Upregulated in Huang *et al*. (15); ^c^Upregulated in Belling *et al*. (14); DETs: differentially expressed transcripts

**Supplementary Table S4: GENCODE transcript biotypes of DETs in the adult KS testis**

| **GENCODE transcript biotype** | **Upregulated transcripts** | **Hypergeometric p-value** | **Downregulated transcripts** | **Hypergeometric p-value** | **Transcripts detected in the whole dataset** | **GENCODE description** |
| --- | --- | --- | --- | --- | --- | --- |
| antisense | 13 | 0.78 | 21 | **3.3E-5** | 5,669 | Have transcripts that overlap the genomic span (i.e. exon or introns) of a protein-coding locus on the opposite strand |
| lincRNA | 36 | **0.0018** | 4 | NC | 7,645 | Long, intervening non-coding RNAs that can be found in evolutionarily conserved, intergenic regions |
| Nonsense-mediated decay (NMD) | 22 | 0.29 | 9 | NC | 6,919 | If the coding sequence (following the appropriate reference) of a transcript finishes >50 bp from a downstream splice site, then it is tagged as NMD. If the variant does not cover the full reference-coding sequence, then it is annotated as NMD if NMD is unavoidable, i.e. no matter what the exon structure of the missing portion is, the transcript will be subject to NMD |
| Processed transcript | 41 | 0.076 | 21 | 0.13 | 11,486 | Transcripts that do not contain an ORF |
| Protein-coding | 75 | **0.0059** | 30 | 0.42 | 19,867 | Transcripts that contain an ORF |
| Retained intron | 34 | 0.11 | 15 | 0.36 | 9,649 | Alternatively spliced transcripts believed to contain intronic sequences relative to other coding variants |

NC: Not calculated as the number of transcripts is below 10, ORF: open reading frame

**Supplementary Table S5: Upregulated transcripts in pre-pubertal KS versus controls**

| **Gene symbol** | **Synonyms(s)** | **ENSG** | **Location** | **logFC** | **logCPM** | **p-value** | **XCI** | **RNA biotype** |
| --- | --- | --- | --- | --- | --- | --- | --- | --- |
| RP11-69H7.2 | AC113208.3 | ENSG00000260660 | 15q24.2 | 7.5755 | 8.6834 | 0.0010 |  | Processed transcript, transcribed unitary pseudogene |
| C17orf77 | FLJ31882 | ENSG00000182352 | 17q25.1 | 7.3270 | 9.6888 | 0.0015 |  | Antisense |
| AGPAT3 | LPAAT-gamma | ENSG00000160216 | 21q22.3 | 7.1014 | 9.4547 | 0.0025 |  | Protein-coding, processed transcript, retained intron |
| JAK3 | JAK-3, LJAK, JAKL | ENSG00000105639 | 19p13.11 | 6.9913 | 9.3383 | 0.0025 |  | Protein-coding, processed transcript, retained intron |
| GUSBP2 | SMAC3L, GUSBL1 | ENSG00000241549 | 6p22.2 | 6.9979 | 9.3449 | 0.0028 |  | Processed transcript, transcribed unprocessed pseudogene |
| XIST | LINC00001 | ENSG00000229807 | Xq13.2 | 10.022 | 6.1989 | 0.0031 | Mostly S | lincRNA |
| MRPS22 | C3orf5, GK002, MRP-S22, GIBT | ENSG00000175110 | 3q23 | 6.7488 | 9.0833 | 0.0034 |  | Protein-coding, processed transcript, retained intron, nonsense-mediated decay |
| KIF26B | FLJ10157 | ENSG00000162849 | 1q44 | 7.6003 | 7.7228 | 0.0034 |  | Protein-coding, processed transcript, retained intron |
| NBEAP1 | BCL8, BCL8A | ENSG00000258590 | 15q11.2 | 6.7469 | 9.0813 | 0.0037 |  | Retained intron, transcribed unprocessed pseudogene |
| HARS |  | ENSG00000170445 | 15q31.3 | 6.5583 | 8.8821 | 0.0040 |  | Protein-coding, retained intron, nonsense-mediated decay |
| RP11-734K2.4 | POC1B-AS1 | ENSG00000270344 | 12q21.33 | 7.1166 | 9.4690 | 0.0041 |  | Antisense |
| THRAP3P1 | THRAP3L | ENSG00000227339 | 3p23 | 7.0608 | 9.4106 | 0.0046 |  | Processed pseudogene |
| LRP11 | MANSC3 | ENSG00000120256 | 6q25.1 | 7.0362 | 9.3850 | 0.0048 |  | Protein-coding, processed transcript |
| MR1 | HLALS | ENSG00000153029 | 1q25.3 | 7.0245 | 9.3729 | 0.0050 |  | Protein-coding, processed transcript, retained intron |
| SIRT3 | SIR2L3 | ENSG00000142082 | 11p15.5 | 7.0001 | 9.3471 | 0.0051 |  | Protein-coding, processed transcript, retained intron, nonsense-mediated decay |
| EFS | CASS3, EFS1, SIN | ENSG00000100842 | 14q11.2 | 6.0650 | 8.3485 | 0.0055 |  | Protein-coding |
| CEP135 | KIAA0635, CEP4 | ENSG00000174799 | 4q12 | 6.9254 | 9.2689 | 0.0057 |  | Protein-coding, retained intron |
| GOLGA1 | MGC33154 | ENSG00000136935 | 9q33.3 | 6.9409 | 9.2852 | 0.0058 |  | Protein-coding, retained intron, nonsense-mediated decay |
| YTHDF3 | FLJ31657 | ENSG00000185728 | 8q12.3 | 6.9692 | 9.3166 | 0.0059 |  | Protein-coding, processed transcript, retained intron, nonsense-mediated decay |
| RP5-1039K5.16 | AL031587.1 | ENSG00000222044 | 22q13.1 | 6.8453 | 9.1851 | 0.0065 |  | Antisense |
| RP11-1069G10.1 | AC103740.1 | ENSG00000259370 | 15q22.2 | 4.8459 | 6.9375 | 0.0067 |  | Antisense |
| TRMT5 | KIAA1393,  TRM5 | ENSG00000126814 | 14q23.1 | 7.0506 | 9.3997 | 0.0069 |  | Protein-coding |
| FBXL18 | Fbl18 | ENSG00000155034 | 7q22.1 | 6.5904 | 8.9143 | 0.0073 |  | Protein-coding, nonsense-mediated decay |
| DRAM1 | DRAM | ENSG00000136048 | 12q23.2 | 6.8789 | 9.2203 | 0.0075 |  | Protein-coding, retained intron, nonsense-mediated decay |
| USP20 | KIAA1003 | ENSG00000136878 | 9q34.11 | 6.7419 | 9.0756 | 0.0076 |  | Protein-coding, processed transcript |
| RNPEPL1 |  | ENSG00000142327 | 2q37.3 | 7.2825 | 8.4220 | 0.0077 |  | Protein-coding, processed transcript, retained intron |
| MTF2 | TDRD19A, M96, PCL2 | ENSG00000143033 | 1p22.1 | 6.8498 | 9.1903 | 0.0078 |  | Protein-coding, processed transcript, retained intron |
| ENDOV |  | ENSG00000173818 | 17q25.3 | 6.7280 | 9.0609 | 0.0080 |  | Protein-coding, processed transcript, retained intron, nonsense-mediated decay |
| ALPL | TNSALP, HOPS | ENSG00000162551 | 1p36.12 | 6.6845 | 9.0153 | 0.0084 |  | Protein-coding, processed transcript |
| GS1-519E5.1 | AC003684.1 | ENSG00000228543 | Xp22.31 | 6.7043 | 9.0379 | 0.0085 |  | lincRNA |
| ZDHHC18 |  | ENSG00000204160 | 1p36.11 | 6.9640 | 9.3120 | 0.0085 |  | Protein-coding, processed transcript |
| CDC20 | CDC20A, p55CDC | ENSG00000117399 | 1p34.2 | 6.6444 | 8.9727 | 0.0086 |  | Protein-coding, processed transcript |
| HSD17B12 | SDR12C1, KAR | ENSG00000149084 | 11p11.2 | 6.6618 | 8.9927 | 0.0088 |  | Protein-coding, processed transcript, retained intron, nonsense-mediated decay |
| DECR1 | SDR18C1, DECR | ENSG00000104325 | 8q21.3 | 6.6240 | 8.9519 | 0.0089 |  | Protein-coding, processed transcript, retained intron, nonsense-mediated decay |
| GPRASP1 | GASP, GASP1 | ENSG00000198932 | Xq22.1 | 6.6795 | 9.0115 | 0.0091 | Mostly S | Protein-coding, retained intron |
| HTR5BP | HTR5B, 5-HT5B, GPR134 | ENSG00000125631 | 2q14.1 | 6.5732 | 8.8963 | 0.0092 |  | Processed transcript, transcribed unprocessed pseudogene |
| ZNF800 |  | ENSG00000048405 | 7q31.33 | 6.8763 | 9.2194 | 0.0092 |  | Protein-coding, processed transcript |
| PKD1L2 | KIAA1879 | ENSG00000166473 | 16q23.2 | 7.7349 | 8.2921 | 0.0093 |  | Protein-coding, processed transcript, retained intron, polymorphic pseudogene |
| BEX2 | DJ79P11.1 | ENSG00000133134 | Xq22.2 | 6.4813 | 8.7983 | 0.0097 | Mostly S | Protein-coding |
| ST14 | HAI, SNC19 | ENSG00000149418 | 11q24.3 | 5.1516 | 7.3148 | 0.0097 |  | Protein-coding, retained intron |
| SCN2B |  | ENSG00000149575 | 11q23.3 | 6.8215 | 9.1618 | 0.0098 |  | Protein-coding |
| GAB3 |  | ENSG00000160219 | Xq28 | 4.9000 | 7.0046 | 0.0100 | Mostly S | Protein-coding, processed transcript |

XCI: X‑chromosome inactivation status (25); S: silenced.

**Supplementary Table S6: Downregulated transcripts in pre-pubertal KS versus controls**

| **Gene symbol** | **Synonym(s)** | **ENSG** | **Location** | **logFC** | **logCPM** | **p-value** | **XCI** | **RNA biotype** |
| --- | --- | --- | --- | --- | --- | --- | --- | --- |
| SASH1 | KIAA0790, SH3D6A | ENSG00000111961 | 6q24.3-q25.1 | -10.479 | 8.0957 | 0.0005 |  | Protein-coding, processed transcript, retained intron, nonsense-mediated decay |
| ULK1 | ATG1A, ATG1 | ENSG00000177169 | 12q24.33 | -9.8746 | 7.5073 | 0.0008 |  | Protein-coding, retained intron |
| HOOK3 | HK3 | ENSG00000168172 | 8p11.21 | -9.7638 | 7.4007 | 0.0009 |  | Protein-coding, processed transcript, retained intron, nonsense-mediated decay |
| PDCD6IP | AIP1, Hp95 | ENSG00000170248 | 3p22.3 | -10.202 | 7.8239 | 0.0011 |  | Protein-coding, processed transcript, retained intron, nonsense-mediated decay |
| SCART1 | CD163c-alpha | ENSG00000214279 | 10q26.3 | -9.8941 | 7.5254 | 0.0012 |  | Protein-coding, retained intron |
| ULK2 | ATG1B | ENSG00000083290 | 17p11.2 | -9.7473 | 7.3832 | 0.0014 |  | Protein-coding, processed transcript, retained intron, nonsense-mediated decay |
| DEPDC5 | DEP.5 | ENSG00000100150 | 22q12.2-q12.3 | -9.4672 | 7.1149 | 0.0016 |  | Protein-coding, processed transcript, retained intron, nonsense-mediated decay |
| EIF2B5 | EIF-2B | ENSG00000145191 | 3q27.1 | -9.3936 | 7.0442 | 0.0017 |  | Protein-coding, processed transcript, retained intron, nonsense-mediated decay |
| STARD7 | GTT1 | ENSG00000084090 | 2q11.2 | -9.3749 | 7.0263 | 0.0019 |  | Protein-coding, processed transcript, retained intron |
| TRIP11 | CEV14, GMAP210, Trip230 | ENSG00000100815 | 14q32.12 | -9.8968 | 7.5294 | 0.0020 |  | Protein-coding, processed transcript, nonsense-mediated decay |
| TOMM70 |  | ENSG00000154174 | 3q12.2 | -9.3281 | 6.9815 | 0.0021 |  | Protein-coding, retained intron |
| MCF2 | ARHGEF21, DBL | ENSG00000101977 | Xq27.1 | -9.5819 | 7.2237 | 0.0021 | Mostly S | Protein-coding, processed transcript |
| GLTSCR1L | KIAA0240 | ENSG00000112624 | 6p21.1 | -9.4089 | 7.0592 | 0.0022 |  | Protein-coding |
| KMT5B | CGI-85, SUV420H1 | ENSG00000110066 | 11q13.2 | -9.5389 | 7.1830 | 0.0023 |  | Protein-coding, processed transcript, retained intron, nonsense-mediated decay |
| PIWIL2 | HILI, CT80 | ENSG00000197181 | 8p21.3 | -9.8250 | 7.4582 | 0.0025 |  | Protein-coding, retained intron |
| NSG1 | AC110814.1 | ENSG00000168824 | 4p16.3 | -9.8134 | 7.4469 | 0.0026 |  | Protein-coding, processed transcript, retained intron, nonsense-mediated decay |
| UBE3C |  | ENSG00000009335 | 7q36.3 | -9.1810 | 6.8412 | 0.0026 |  | Protein-coding, retained intron, nonsense-mediated decay |
| COBLL1 | KIAA0977 | ENSG00000082438 | 2q24.3 | -10.134 | 7.7578 | 0.0027 |  | Protein-coding, processed transcript, retained intron, nonsense-mediated decay |
| RP11-448A19.1 | AC078846.1 | ENSG00000273329 | 7q32.2 | -9.2356 | 6.8935 | 0.0028 |  | lincRNA |
| ZNF134 | pHZ-15 | ENSG00000213762 | 19q13.43 | -9.1793 | 6.8401 | 0.0030 |  | Protein-coding, processed transcript |
| DDX52 | ROK1 | ENSG00000278053 | 17q12 | -9.2160 | 6.8756 | 0.0031 |  | Protein-coding, retained intron, nonsense-mediated decay |
| GABRB2 |  | ENSG00000145864 | 5q34 | -8.0175 | 7.4137 | 0.0031 |  | Protein-coding, processed transcript, retained intron |
| TAPT1 | FLJ90013 | ENSG00000169762 | 4p15.32 | -9.2546 | 6.9125 | 0.0032 |  | Protein-coding, processed transcript, retained intron, nonsense-mediated decay |
| FAM19A2 | TAFA2, TAFA-2 | ENSG00000198673 | 12q14.1 | -9.1629 | 6.8241 | 0.0034 |  | Protein-coding, processed transcript, retained intron, nonsense-mediated decay |
| TUBGCP6 | GCP6, KIAA1669, DJ402G11.6 | ENSG00000128159 | 22q13.33 | -9.6540 | 7.2940 | 0.0034 |  | Protein-coding, processed transcript, retained intron |
| THAP9 | FLJ34093 | ENSG00000168152 | 4q21.22 | -9.6281 | 7.2695 | 0.0034 |  | Protein-coding, nonsense-mediated decay |
| TUB | rd5 | ENSG00000166402 | 11q15.4 | -9.1133 | 6.7764 | 0.0036 |  | Protein-coding |
| USH1C | NY-CO-38,  PDZ-73, DFNB18, PDZ73 | ENSG00000006611 | 11p15.1 | -9.6504 | 7.2901 | 0.0037 |  | Protein-coding, processed transcript, retained intron, nonsense-mediated decay, TEC |
| ZNF317 |  | ENSG00000130803 | 19p13.2 | -9.2620 | 6.9186 | 0.0037 |  | Protein-coding, retained intron, nonsense-mediated decay |
| CSTF3 | CstF-77 | ENSG00000176102 | 11p13 | -9.5827 | 7.2263 | 0.0038 |  | Protein-coding, processed transcript, nonsense-mediated decay |
| DPYD | DPD | ENSG00000188641 | 1p21.3 | -9.0497 | 6.7170 | 0.0041 |  | Protein-coding, processed transcript, retained intron |
| NAB1 |  | ENSG00000138386 | 2q32.2 | -9.2071 | 6.8680 | 0.0042 |  | Protein-coding, processed transcript |
| SEMA3C | SEMAE | ENSG00000075223 | 7q21.11 | -9.5083 | 7.1540 | 0.0043 |  | Protein-coding, processed transcript, nonsense-mediated decay |
| XIRP2 | CMYA3 | ENSG00000163092 | 2q24.3 | -8.8241 | 6.5064 | 0.0046 |  | Protein-coding |
| MGAT4C | HGNT-IV-H | ENSG00000182050 | 12q21.31-q21.32 | -8.3180 | 7.7074 | 0.0046 |  | Protein-coding, processed transcript |
| BMP2K |  | ENSG00000138756 | 4q21.21 | -8.9019 | 6.5786 | 0.0047 |  | Protein-coding, retained intron, nonsense-mediated decay |
| PHLPP2 | PPM3B, PHLPPL, KIAA0931 | ENSG00000040199 | 16q22.2 | -8.1281 | 7.2547 | 0.0048 |  | Protein-coding, retained intron, nonsense-mediated decay |
| DNTTIP2 | HSU15552, ERBP, TdIF2 | ENSG00000067334 | 1p22.1 | -9.4735 | 7.1198 | 0.0049 |  | Protein-coding, processed transcript, retained intron, nonsense-mediated decay |
| PSMG4 | PAC4, C6orf86 | ENSG00000180822 | 6p25.2 | -8.9911 | 0.6662 | 0.0049 |  | Protein-coding, processed transcript, nonsense-mediated decay |
| JADE2 | PHF15, KIAA0239, JADE‑2 | ENSG00000043143 | 5q31.1 | -8.2172 | 7.3383 | 0.0049 |  | Protein-coding, processed transcript, retained intron, nonsense-mediated decay |
| KLHL3 | KIAA1129 | ENSG00000146021 | 5q31.1 | -9.6787 | 7.3161 | 0.0050 |  | Protein-coding, processed transcript, retained intron, nonsense-mediated decay |
| GAS5 | SNHG2 | ENSG00000234741 | 1q25.1 | -8.9986 | 6.6689 | 0.0050 |  | lincRNA, retained intron |
| PRKG1 | PRKG1B, PKG, PRKGR1B, PGK | ENSG00000185532 | 10q11.23-q21.1 | -9.5472 | 7.1897 | 0.0052 |  | Protein-coding, processed transcript |
| PSME4 | KIAA0077, PA200 | ENSG00000068878 | 2p16.2 | -9.3970 | 7.0489 | 0.0053 |  | Protein-coding, processed transcript, retained intron, nonsense-mediated decay |
| RBBP8 | SCKL2, RIM, COM1, CtIP | ENSG00000101773 | 18q11.2 | -8.8356 | 6.5172 | 0.0053 |  | Protein-coding, processed transcript, retained intron, nonsense-mediated decay |
| C5 | CPAMD4, C5b, C5a | ENSG00000106804 | 9q33.2 | -9.4356 | 7.0831 | 0.0055 |  | Protein-coding, processed transcript, retained intron |
| UBE2A | HHR6A, RAD6A, UBC2 | ENSG00000077721 | Xq24 | -9.6190 | 7.2586 | 0.0055 | Mostly S | Protein-coding, retained intron, nonsense-mediated decay |
| CARD14 | PSORS2, BIMP2, CARMA2 | ENSG00000141527 | 17q25.3 | -9.4133 | 7.0620 | 0.0056 |  | Protein-coding, processed transcript, retained intron, nonsense-mediated decay |
| ATP10A | ATPVC ATP10C, KIAA0566 | ENSG00000206190 | 15q12 | -9.3418 | 6.9948 | 0.0057 |  | Protein-coding, processed transcript, retained intron, nonsense-mediated decay |
| GCFC2 | DNABF, C2orf3, GCF, TCF9 | ENSG00000005436 | 2p12 | -9.0308 | 0.6699 | 0.0057 |  | Protein-coding, retained intron, nonsense-mediated decay |
| LRGUK | FLJ32786, CFAP246 | ENSG00000155530 | 7q33 | -8.8547 | 6.5331 | 0.0057 |  | Protein-coding, processed transcript |
| FGD4 | FRABP, ZFYVE6, CMT4H | ENSG00000139132 | 12p11.21 | -7.9596 | 7.3098 | 0.0058 |  | Protein-coding, processed transcript, retained intron, nonsense-mediated decay |
| ATAD2B | KIAA1240 | ENSG00000119778 | 2p24.1-p23.3 | -8.4181 | 0.0613 | 0.0059 |  | Protein-coding, processed transcript, retained intron |
| KCMF1 | PCMF | ENSG00000176407 | 2p11.2 | -8.5594 | 6.2581 | 0.0059 |  | Protein-coding |
| PURB | PURBETA | ENSG00000146676 | 7p13 | -9.3169 | 6.9712 | 0.0060 |  | Protein-coding |
| NGLY1 | PNG1, FLJ11005 | ENSG00000151092 | 3p24.2 | -9.4479 | 7.0961 | 0.0060 |  | Protein-coding, processed transcript, retained intron, nonsense-mediated decay |
| FERMT1 | URP1, KIND1, C20orf42 | ENSG00000101311 | 20p12.3 | -8.8862 | 6.5627 | 0.0061 |  | Protein-coding, processed transcript |
| LTA4H |  | ENSG00000111144 | 12q23.1 | -9.2833 | 6.9398 | 0.0061 |  | Protein-coding, processed transcript, retained intron, nonsense-mediated decay |
| CTD-2516F10.2 | AC107884.1 | ENSG00000251364 | 11p15.4 | -8.5309 | 6.2343 | 0.0062 |  | Antisense |
| SMOC1 |  | ENSG00000198732 | 11q24.2 | -9.4125 | 7.0613 | 0.0062 |  | Protein-coding, processed transcript, retained intron |
| TRAPPC13 | C5orf44 | ENSG00000113597 | 5q12.3 | -7.2348 | 5.0884 | 0.0063 |  | Protein-coding, retained intron, nonsense-mediated decay |
| NUP50 | NPAP60L | ENSG00000093000 | 22q13.31 | -9.2984 | 6.9526 | 0.0064 |  | Protein-coding, processed transcript, retained intron, nonsense-mediated decay |
| SESTD1 |  | ENSG00000187231 | 2q31.2 | -9.2603 | 6.9185 | 0.0064 |  | Protein-coding, processed transcript, retained intron, nonsense-mediated decay |
| MYO3B |  | ENSG00000071909 | 2q31.1 | -8.4219 | 6.1347 | 0.0064 |  | Protein-coding, processed transcript, retained intron, nonsense-mediated decay |
| CHD5 |  | ENSG00000116254 | 1p36.31 | -8.8297 | 6.5101 | 0.0064 |  | Protein-coding, nonsense-mediated decay |
| FAM168B | MANI | ENSG00000152102 | 2q21.1 | -8.6701 | 6.3634 | 0.0064 |  | Protein-coding |
| ATRNL1 | KIAA0534, ALP | ENSG00000107518 | 10q25.3 | -7.3879 | 7.4951 | 0.0065 |  | Protein-coding, processed transcript, nonsense-mediated decay |
| SHQ1 | FLJ10539 | ENSG00000144736 | 3p13 | -8.1224 | 5.8617 | 0.0067 |  | Protein-coding, processed transcript, nonsense-mediated decay |
| THADA | GITA, KIAA1767, ARMC13 | ENSG00000115970 | 2p21 | -9.4204 | 7.0723 | 0.0067 |  | Protein-coding, processed transcript, retained intron, nonsense-mediated decay |
| E2F7 |  | ENSG00000165891 | 12q21.2 | -9.2048 | 6.8649 | 0.0069 |  | Protein-coding, retained intron, nonsense-mediated decay |
| SNAP47 | SVAP1, SNAP-47, C1orf142 | ENSG00000143740 | 1q42.13 | -8.4217 | 0.6134 | 0.0069 |  | Protein-coding, processed transcript |
| YWHAE | FLJ45465 | ENSG00000108953 | 17p13.3 | -8.9403 | 6.6163 | 0.0071 |  | Protein-coding, processed transcript, retained intron, nonsense-mediated decay |
| DPY19L4 |  | ENSG00000156162 | 8q22.1 | -9.3279 | 6.9810 | 0.0071 |  | Protein-coding, nonsense-mediated decay |
| AKAP9 | YOTIAO, PRKA9, AKAP350, LQT11, PPP1R45 | ENSG00000127914 | 7q21.2 | -6.8451 | 7.9473 | 0.0071 |  | Protein-coding, processed transcript, retained intron |
| OCRL | OCRL1 | ENSG00000122126 | Xq26.1 | -7.8373 | 7.1934 | 0.0072 | Mostly S | Protein-coding, processed transcript |
| TACR1 | SPR, NKIR, NK1R, TAC1R | ENSG00000115353 | 2p12 | -9.2520 | 6.9079 | 0.0072 |  | Protein-coding, processed transcript |
| GTDC1 | FLJ11753 | ENSG00000121964 | 2q22.3 | -9.2488 | 6.9049 | 0.0072 |  | Protein-coding, processed transcript, retained intron, nonsense-mediated decay |
| PACRG-AS3 |  | ENSG00000225683 | 6q26 | -8.6328 | 0.6325 | 0.0072 |  | Antisense |
| CDR2 | CDR62 | ENSG00000140743 | 16p12.2 | -9.3072 | 6.9609 | 0.0072 |  | Protein-coding, processed transcript, nonsense-mediated decay |
| CTC-360P9.4 | LINC01837 | ENSG00000267489 | 19q12-q13.11 | -7.8830 | 0.5651 | 0.0072 |  | lincRNA |
| CTD-2384A14.1 | LINC02327 | ENSG00000258038 | 14q12 | -9.4270 | 7.0748 | 0.0073 |  | lincRNA |
| SLC5A1 | SGLT1, NAGT | ENSG00000100170 | 22q12.3 | -8.8869 | 6.5663 | 0.0073 |  | Protein-coding, retained intron |
| FAM76B | MGC33371 | ENSG00000077458 | 11q21 | -8.6664 | 6.3558 | 0.0073 |  | Protein-coding, processed transcript, retained intron, nonsense-mediated decay |
| NAMPT | PBEF1, PBEF | ENSG00000105835 | 7q22.3 | -7.5597 | 7.5512 | 0.0073 |  | Protein-coding, processed transcript, retained intron |
| SLTM | FLJ13213 | ENSG00000137776 | 15q22.1 | -9.3926 | 7.0412 | 0.0073 |  | Protein-coding, processed transcript, retained intron, nonsense-mediated decay |
| GANC |  | ENSG00000214013 | 15q15.1 | -7.7515 | 5.5355 | 0.0075 |  | Protein-coding, processed transcript, retained intron, nonsense-mediated decay |
| RRP1B | RRP1, KIAA0179 | ENSG00000160208 | 21q22.3 | -7.9747 | 7.3766 | 0.0075 |  | Protein-coding, retained intron |
| COPB1 | COPB | ENSG00000129083 | 11p15.2 | -9.2115 | 6.8697 | 0.0076 |  | Protein-coding, processed transcript |
| ALOX5AP | FLAP | ENSG00000132965 | 13q12.3 | -9.2180 | 6.8756 | 0.0076 |  | Protein-coding, processed transcript |
| SEPT10 | FLJ11619 | ENSG00000186522 | 2q13 | -9.1134 | 6.7787 | 0.0076 |  | Protein-coding, processed transcript, retained intron, nonsense-mediated decay |
| RP11-358D17.2 | AC096745.2 | ENSG00000280551 | 4q26 | -9.1501 | 0.0681 | 0.0077 |  | TEC |
| C19orf66 | FLJ11286, IRAV | ENSG00000130813 | 19p13.2 | -7.8721 | 7.2256 | 0.0078 |  | Protein-coding, retained intron, nonsense-mediated decay |
| LCLAT1 | LYCAT, AGPAT8, FLJ37965, ALCAT1 | ENSG00000172954 | 2p23.1 | -8.6679 | 6.3583 | 0.0078 |  | Protein-coding, processed transcript, nonsense-mediated decay |
| VPS29 | PEP11, DC7, DC15 | ENSG00000111237 | 12q24.11 | -9.1898 | 6.8506 | 0.0079 |  | Protein-coding, processed transcript, retained intron |
| ABR | MDB | ENSG00000159842 | 17p13.3 | -9.0389 | 6.7084 | 0.0080 |  | Protein-coding, processed transcript, retained intron, nonsense-mediated decay |
| RPS6KA6 | RSK4 | ENSG00000072133 | Xq21.1 | -8.2300 | 0.5962 | 0.0080 | Mostly S | Protein-coding, processed transcript |
| CLCN6 | CLC-6, KIAA0046 | ENSG00000011021 | 1p36.22 | -8.8033 | 6.4851 | 0.0081 |  | Protein-coding, processed transcript |
| NUMB | C14orf41 | ENSG00000133961 | 14q24.2-q24.3 | -6.9264 | 4.8366 | 0.0082 |  | Protein-coding, processed transcript, retained intron |
| H1F0 | H1FV, H10 | ENSG00000189060 | 22q13.1 | -7.8497 | 7.6288 | 0.0082 |  | Protein-coding |
| NCOA4 | PTC3, ARA70, ELE1, RFG | ENSG00000266412 | 10q11.22 | -9.0909 | 6.7564 | 0.0082 |  | Protein-coding |
| DCLRE1C | SCIDA, ARTEMIS | ENSG00000152457 | 10p13 | -7.6365 | 0.5435 | 0.0083 |  | Protein-coding, processed transcript |
| NUDT21 | CPSF5, CFIM25 | ENSG00000167005 | 16q13 | -8.0984 | 7.4981 | 0.0084 |  | Protein-coding, retained intron |
| SUSD6 | KIAA0247 | ENSG00000100647 | 14q24.1 | -8.7263 | 6.4111 | 0.0085 |  | Protein-coding, processed transcript, retained intron |
| CKAP2 | TMAP, LB1, FLJ10749 | ENSG00000136108 | 13q14.3 | -8.9003 | 6.5755 | 0.0085 |  | Protein-coding, retained intron |
| RNF13 | RZF | ENSG00000082996 | 3q25.1 | -7.3398 | 0.0518 | 0.0085 |  | Protein-coding, retained intron, nonsense-mediated decay |
| MYO1B |  | ENSG00000128641 | 2q32.3 | -7.6100 | 7.1624 | 0.0085 |  | Protein-coding, processed transcript, retained intron |
| RP11-498C9.15 | AC145207.5 | ENSG00000263731 | 17q25.3 | -9.0328 | 6.7027 | 0.0085 |  | lincRNA |
| ATP8A1 | ATPIA | ENSG00000124406 | 4p13 | -8.8833 | 6.5598 | 0.0086 |  | Protein-coding, retained intron, nonsense-mediated decay |
| LCA5L | MGC33295, C21orf13 | ENSG00000157578 | 21q22.2 | -9.2391 | 6.8954 | 0.0086 |  | Protein-coding, processed transcript, retained intron |
| SYTL2 | PPP1R151, SLP2 | ENSG00000137501 | 11q14.1 | -7.4684 | 0.7609 | 0.0086 |  | Protein-coding, processed transcript, retained intron, nonsense-mediated decay |
| TANGO2 | C22orf25 | ENSG00000183597 | 22q11.21 | -9.0861 | 6.7505 | 0.0088 |  | Protein-coding, processed transcript, retained intron, nonsense-mediated decay |
| CHEK2 | PP1425, CDS1, CHK2, RAD53 | ENSG00000183765 | 22q12.1 | -9.0955 | 6.7594 | 0.0088 |  | Protein-coding, retained intron, nonsense-mediated decay |
| SERINC3 | TMS-1, AIGP1, SBBI99, TDE, TDE1, DIFF33 | ENSG00000132824 | 20q13.2 | -9.1320 | 0.6793 | 0.0089 |  | Protein-coding, processed transcript |
| SORT1 | Gp95, NT3 | ENSG00000134243 | 1p13.3 | -9.1448 | 6.8087 | 0.0089 |  | Protein-coding, processed transcript, retained intron, nonsense-mediated decay |
| DHX29 | DDX29 | ENSG00000067248 | 5q11.2 | -9.1143 | 6.7808 | 0.0090 |  | Protein-coding, retained intron |
| ETF1 | ERF, SUP45L1, TB3-1, RF1 | ENSG00000120705 | 5q31.2 | -9.2165 | 6.8761 | 0.0090 |  | Protein-coding, processed transcript, retained intron, nonsense-mediated decay |
| PKP4 |  | ENSG00000144283 | 2q24.1 | -8.9722 | 6.6458 | 0.0090 |  | Protein-coding, processed transcript, retained intron, nonsense-mediated decay |
| LRRC9 |  | ENSG00000131951 | 14q23.1 | -8.6114 | 6.3098 | 0.0091 |  | Protein-coding, processed transcript, retained intron, nonsense-mediated decay |
| RPL13 | L13, BBC1 | ENSG00000167526 | 16q24.3 | -7.7283 | 7.2793 | 0.0092 |  | Protein-coding, retained intron, nonsense-mediated decay |
| SYT12 | SRG1 | ENSG00000173227 | 11q13.2 | -8.9524 | 6.6262 | 0.0093 |  | Protein-coding, processed transcript, retained intron |
| SP3 | SPR-2 | ENSG00000172845 | 2q31.1 | -8.2635 | 5.9926 | 0.0093 |  | Protein-coding, processed transcript, retained intron |
| RABAC1 | YIP3, PRA1, PRAF1 | ENSG00000105404 | 19q13.2 | -8.9837 | 6.6561 | 0.0094 |  | Protein-coding, retained intron |
| KBTBD8 | TA-KRP, KIAA1842 | ENSG00000163376 | 3p14.1 | -9.1501 | 6.8145 | 0.0094 |  | Protein-coding, processed transcript |
| TNFRSF19 | TAJ, TROY, TRADE | ENSG00000127863 | 13q12.12 | -9.0987 | 0.6762 | 0.0094 |  | Protein-coding, processed transcript |
| NENF | SCIRP10, CIR2, SPUF | ENSG00000117691 | 1q32.3 | -7.9373 | 5.6997 | 0.0094 |  | Protein-coding, processed transcript |
| METTL16 | METT10D, MGC3329 | ENSG00000127804 | 17p13.3 | -7.7176 | 5.5069 | 0.0095 |  | Protein-coding, processed transcript, retained intron, nonsense-mediated decay |
| ARHGAP42 | GRAF3, FLJ32810 | ENSG00000165895 | 11q22.1 | -9.1985 | 6.8560 | 0.0095 |  | Protein-coding, processed transcript, retained intron, nonsense-mediated decay |
| MEF2C |  | ENSG00000081189 | 5q14.3 | -7.6907 | 8.0597 | 0.0095 |  | Protein-coding, processed transcript, retained intron |
| CLASP1 | MAST1, KIAA0622 | ENSG00000074054 | 2q14.2-q14.3 | -8.9457 | 6.6206 | 0.0095 |  | Protein-coding, processed transcript, retained intron |
| BRCC3 | CXorf53, BRCC36 | ENSG00000185515 | Xq28 | -9.0024 | 6.6726 | 0.0095 | Mostly S | Protein-coding, nonsense-mediated decay |
| MOB1A | C2orf6, MOBK1B, FLJ10788 | ENSG00000114978 | 2p13.1 | -7.8900 | 5.6571 | 0.0096 |  | Protein-coding, processed transcript, retained intron |
| FAM117A |  | ENSG00000121104 | 17q21.33 | -8.9972 | 6.6667 | 0.0096 |  | Protein-coding, processed transcript, retained intron, nonsense-mediated decay |
| SLC20A2 | MLVAR, Glvr-2, PiT-2, GLVR2 | ENSG00000168575 | 8p11.21 | -9.0525 | 6.7214 | 0.0096 |  | Protein-coding, processed transcript, retained intron, nonsense-mediated decay |
| LINC01016 |  | ENSG00000249346 | 6p21.31 | -9.0557 | 6.7221 | 0.0098 |  | lincRNA |
| PPP2R2B | SCA12, PR52B, B55beta, PR55-BETA | ENSG00000156475 | 5q32 | -9.1695 | 6.8300 | 0.0098 |  | Protein-coding, processed transcript, nonsense-mediated decay |
| GAPDH | GAPD | ENSG00000111640 | 12p13.31 | -9.1961 | 6.8574 | 0.0098 |  | Protein-coding, retained intron |
| ANGEL1 | Ccr4e, KIAA0759 | ENSG00000013523 | 14q24.3 | -9.0367 | 6.7052 | 0.0099 |  | Protein-coding, processed transcript |
| TSSC1 | EIPR-1 | ENSG00000032389 | 2p25.3 | -9.0279 | 6.6997 | 0.0099 |  | Protein-coding, processed transcript, retained intron, nonsense-mediated decay |
| GLI1 | GLI | ENSG00000111087 | 12q13.3 | -8.1301 | 0.5864 | 0.0100 |  | Protein-coding, retained intron |

XCI: X‑chromosome inactivation status (25); S: silenced.

**Supplementary Table S7: Overlap in developmental differentially expressed transcripts**

| **Gene symbol** | **ENSG** | **Location** | **Fetal** | | **Pre-pubertal** | | **Adult** | | **RNA biotype** |
| --- | --- | --- | --- | --- | --- | --- | --- | --- | --- |
|  |  |  | **logFC** | **p-value** | **logFC** | **p-value** | **logFC** | **p-value** |  |
| XIST | ENSG00000229807 | Xq13.2 | 5.8 | 1.2E-14 | 6.2 | 0.0031 | 11.3 | 1.7E-39 | lincRNA |
| RP11-15H7.2 | ENSG00000205695 | 6q24.1 | 6.7 | 0.0015 |  |  | 9.7 | 0.00062 | Processed pseudogene |
| JSRP1 | ENSG00000167476 | 19p13.3 | -7.0 | 0.0064 |  |  | 9.5 | 0.00093 | Protein-coding, processed transcript |
| RP11-44K6.3 | ENSG00000253939 | 8p11.21 | 6.7 | 0.0038 |  |  | 6.6 | 0.0063 | Antisense |
| RP4-613B23.1 | ENSG00000230084 | 3p22.1 | 7.5 | 0.0063 |  |  | -7.7 | 0.0094 | Antisense |
| JAK3 | ENSG00000229807 | 19p13.11 | -0.098 | 0.90 | 9.3 | 0.0025 | -2.7 | 0.0077 | Protein-coding, processed transcript, retained intron |
| GLI1 | ENSG00000205695 | 12q13.3 | 0.66 | 0.54 | -8.1 | 0.01 | -9.5 | 0.00020 | Protein-coding, retained intron |

**Supplementary Table S8: Antibodies used for immunohistochemical and immunofluorescence staining**

| **Antigen name** | **Source species** | **Unmasking Buffer** | **Dilution (IHC)** | **Dilution (IF)** | **Supplier details** |
| --- | --- | --- | --- | --- | --- |
| AMH | Mouse | TEG buffer (Tris 6.06 g, EGTA 0.95 g in 5 L, pH 9) | 1:200 |  | Gift from R. Cate (Amgen) |
| MAGE-A4 | Mouse | TEG | 1:250 |  | Gift from G.C. Spagnoli (Univ. Hosp. Zurich, Switzerland) |
| Cleaved PARP (Asp214) | Rabbit | 10 mM citrate buffer pH 6 | 1:75 |  | 5625, Cell signaling Technology, Leiden, The Netherlands |
| DACH2 | Rabbit | Citrate buffer (IHC, IF) | 1:600 |  | HPA000258, Sigma-Aldrich, MO, USA |
| FAM9A | Rabbit | TEG buffer (IHC) Citrate buffer (IF) | 1:100 | 1:200 | HPA056076, Sigma-Aldrich, MO, USA |
| INSL3 | Rabbit | Citrate buffer |  | 1:7500 | HPA028615, Atlas Antibodies, Bromma, Sweden |

IHC: Immunohistochemistry; IF: immunofluorescence

**Supplementary Table S9: Samples used for validation of DACH2 and FAM9A**

| **Sample** | **IHC antibodies** | **Fluorescence antibodies** |
| --- | --- | --- |
| aKS1 | DACH2, AMH |  |
| aKS3 | DACH2, FAM9A, AMH | FAM9A, INSL3 |
| aCMC1 | DACH2, FAM9A |  |
| aCMC2 | DACH2, FAM9A |  |
| aCMC3 | DACH2, FAM9A |  |
