# Supplementary material for: Transcriptome analysis of the adult human Klinefelter testis and cellularity-matched controls reveals disturbed differentiation of Sertoli- and Leydig cells
Source: Cell Death Dis. 2018 May 22;9(6):586. doi: 10.1038/s41419-018-0671-1 (PMC5964117; doi:10.1038/s41419-018-0671-1)
Supplement: Supplementary file 2 — Supplementary Figures [file 41419_2018_671_MOESM2_ESM.docx]

**
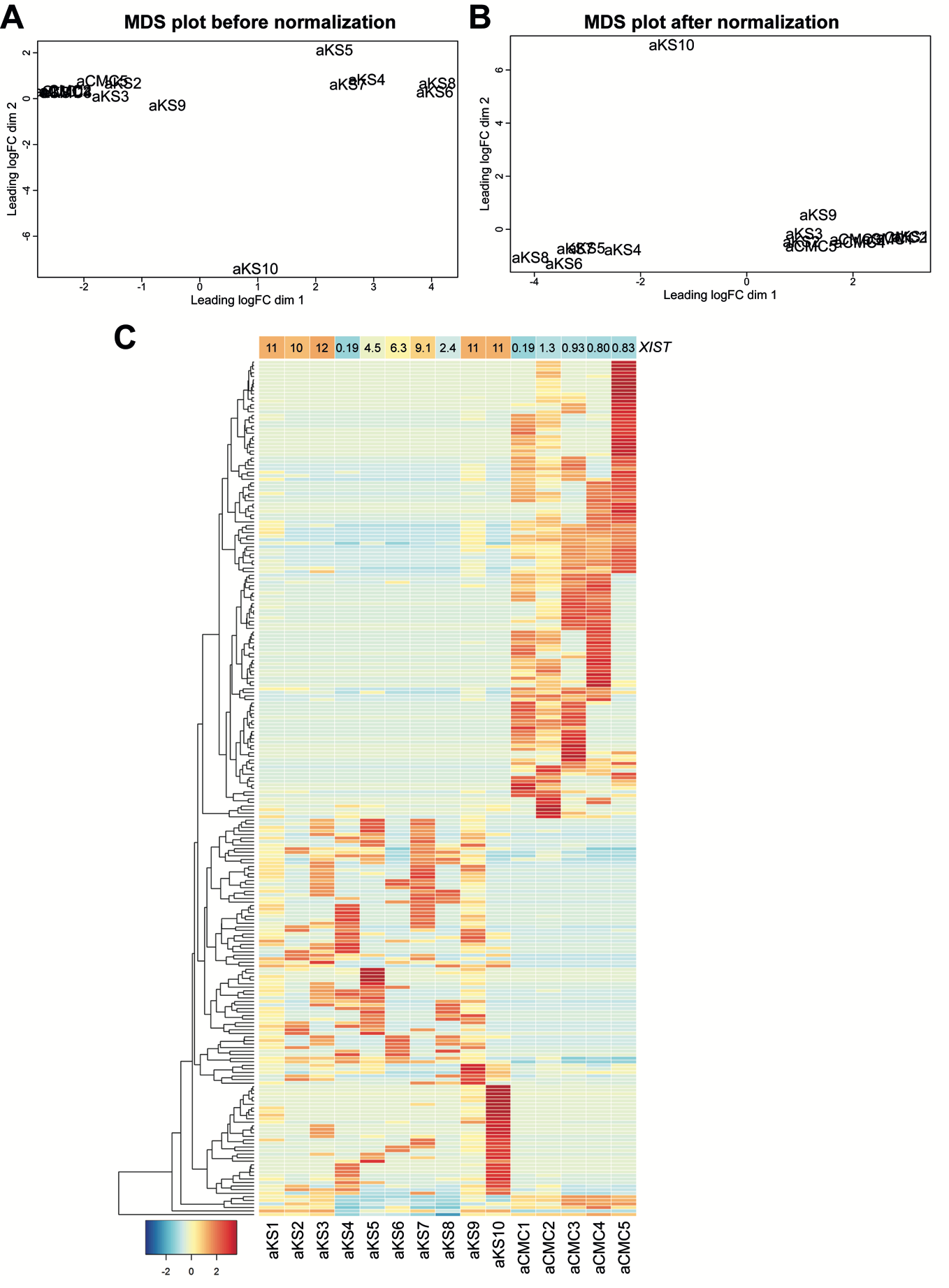
**

**Supplementary Figure S1: Initial MDS plots and heatmap**

Multidimensional scaling (MDS) plot before (**A**) and after (**B)** normalization for library size Notice how the adult Klinefelter syndrome (aKS)10 sample is completely isolated from the rest of the samples. Notice also that a group of five samples: aKS4, aKS5, aKS6, aKS7, and aKS8 are a separate group from the rest of the samples. **C**: Initial heatmap. Notice how different aKS5, aKS6, aKS7 and aKS10 are from the rest of the samples. Expression values for *XIST* are shown for each sample on the top. Notice no upregulation in aKS4 and aKS8.

**
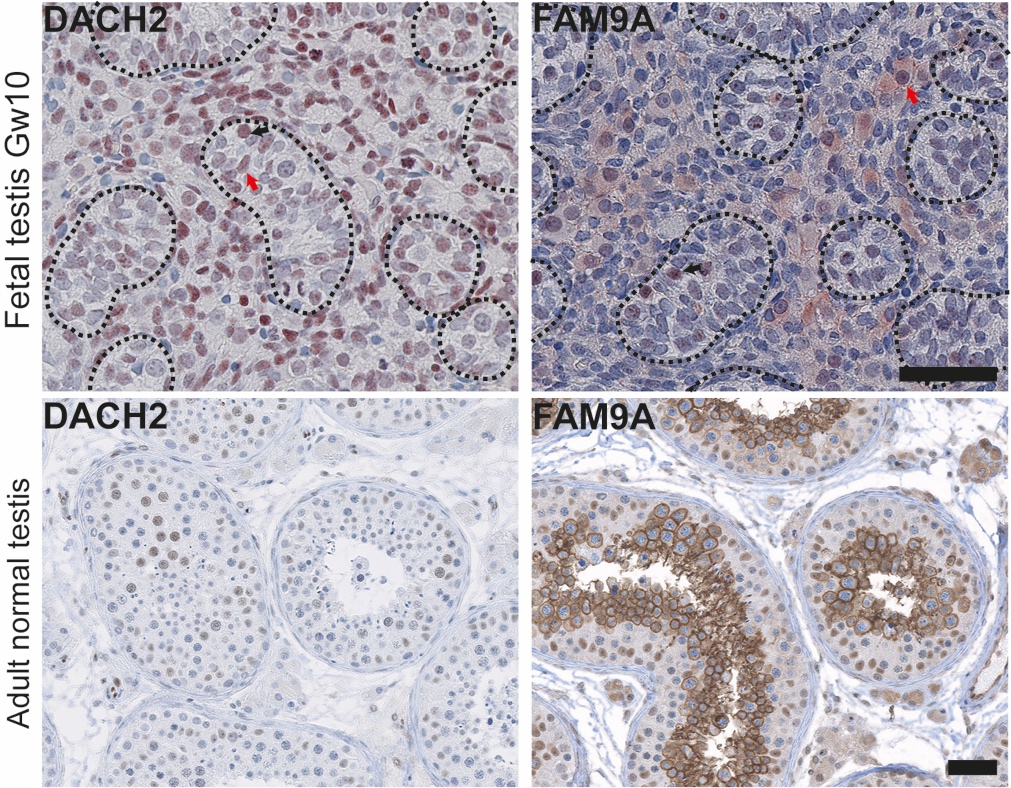
**

**Supplementary Figure S2: Localization of DACH2 and FAM9A in a testis from a fetus aged gestational week (Gw) 10 and in testes with full spermatogenesis**

IHC staining with antibodies against DACH2 and FAM9A of a testis from a fetus aged Gw10 (top) and adult normal testis, i.e. testis with full spermatogenesis (bottom). In the fetal testis (n=1, tubular borders are indicated with dotted lines), DACH2 was expressed in the nuclei of a subpopulation of gonocytes (black arrow, ++), a subpopulation of Sertoli cells (red arrow, +++), interstitial cells with varying intensity (+ to +++), and in the majority of peritubular cells (+++), whereas FAM9A was expressed in the nuclei of a few gonocytes (black arrow, ++) and in the cytoplasm of a subpopulation of interstitial cells (based on morphology mainly Leydig cells) (red arrow, +).

In the normal testis (n=4), DACH2 was expressed in the nuclei of blood vessels (+++), primary spermatocytes (++) and Sertoli cells (+), whereas FAM9A (n=3) was expressed in the cell membrane of later stages of spermatogenesis (including primary spermatocytes and spermatids, +++), in the nuclei of Sertoli cells (++), and in the cytoplasm of Leydig cells with varying intensity (+ to +++). Scale bars correspond to 50 µm.

**
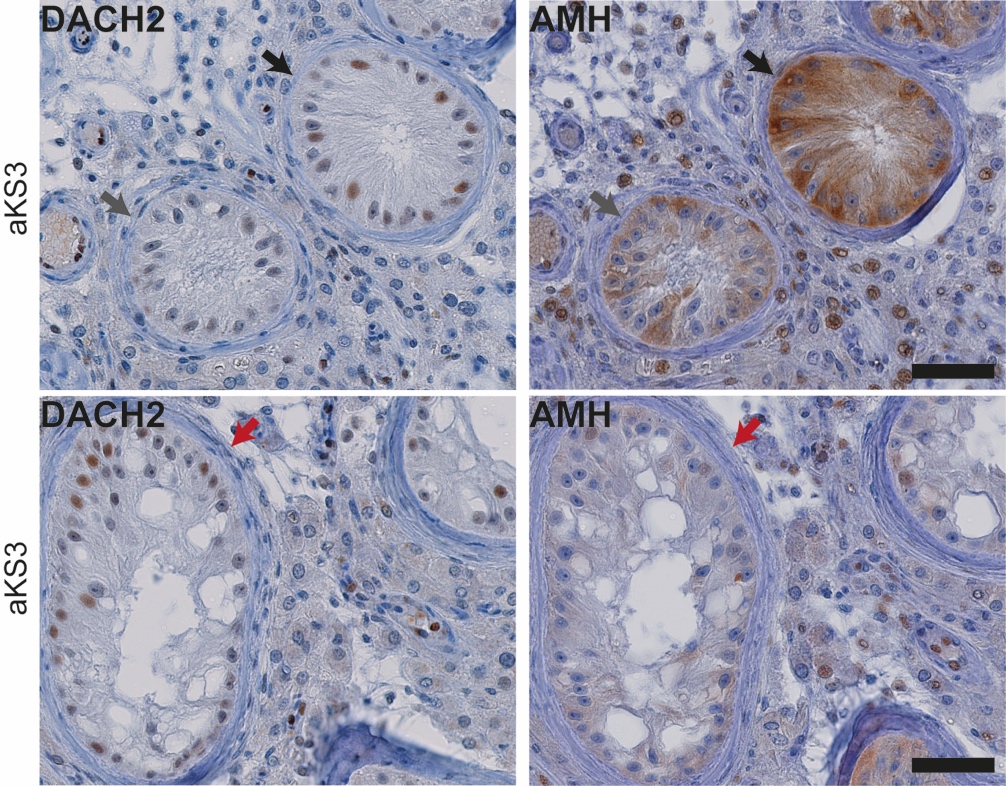
**

**Supplementary Figure S3: Localization of DACH2 and AMH in a testis from an adult man with Klinefelter syndrome (KS)**

IHC staining with antibodies against DACH2 and AMH of a testis from an adult man with KS (which is the same sample as aKS3 used for RNA-sequencing). DACH2 was expressed in most of the nuclei in Sertoli cell-only (SCO) tubules (see also Figure 2). AMH was expressed in the majority of SCO tubules with varying intensity (+ to +++), in Leydig cell nuclei (+++ to negative), and in blood vessels (+++ to negative). The majority of DACH2-positive Sertoli cells also expressed AMH (black arrow). A few tubules contained many DACH2-positive cells, which showed faint to negative staining for AMH (red arrow), but also the opposite was observed. The grey arrow shows a tubule with faintly stained/negative DACH2 Sertoli cells, which is positive for AMH. The same staining patterns were also seen in another KS testis, aKS1, also used for the transcriptome profiles (data not shown). Scale bars correspond to 50 µm.

**
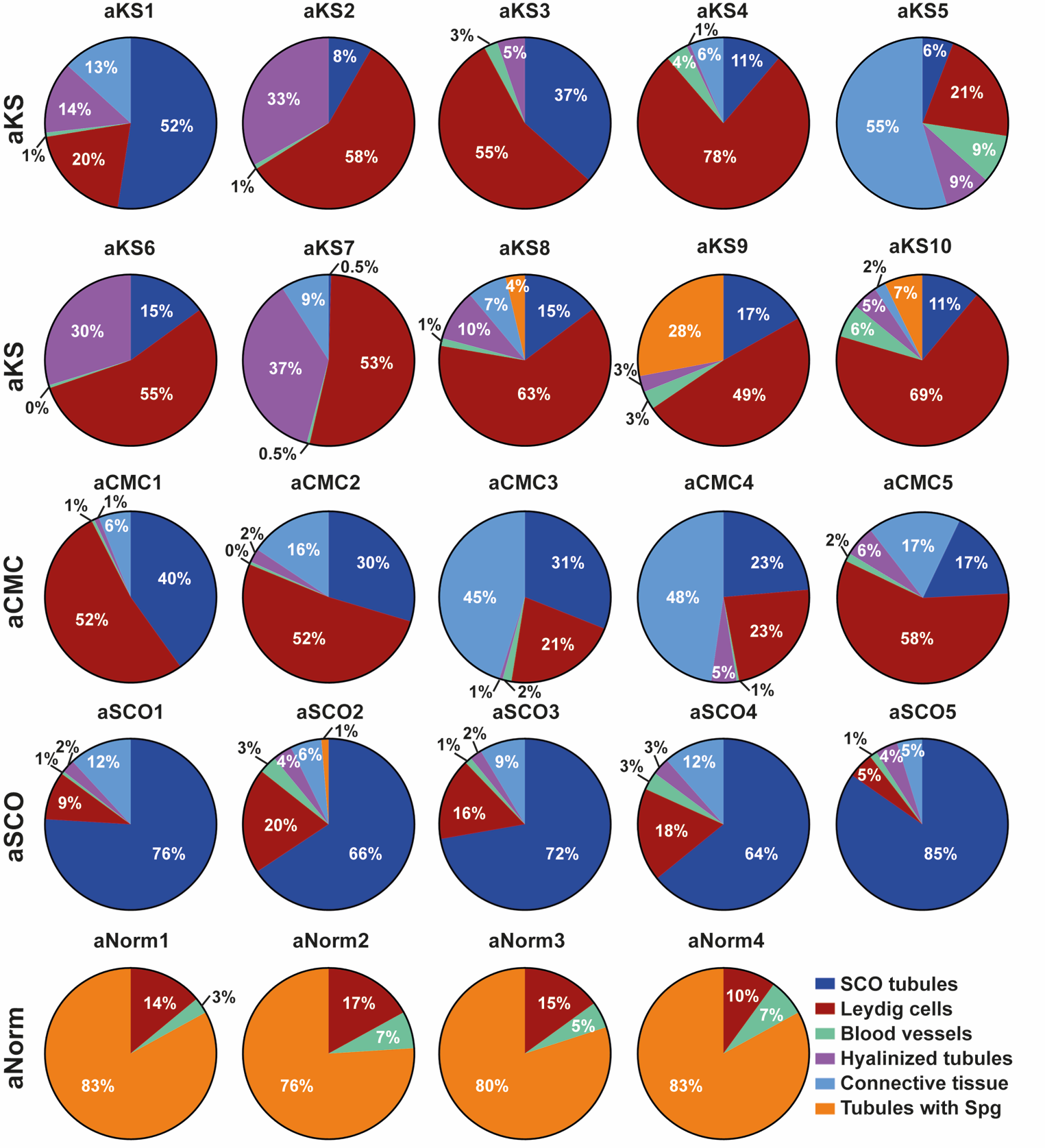
**

**Supplementary Figure S4: Cellularity of all adult samples initially used for RNA-sequencing**

Each testicular biopsy was measured in Adobe Photoshop, and the cellularity was calculated as a percentage of the total area of the sample. Tubules containing spermatogonia (Spg) were evaluated based on MAGE-A4 staining, the rest based on visual inspection. aKS: testes from adult men with Klinefelter syndrome, aCMC: testes from adult men with histology that resembles that of KS. aSCO: testes from adult men with no germ cells in the seminiferous tubules. aNorm: testes with full spermatogenesis from adult men.

**
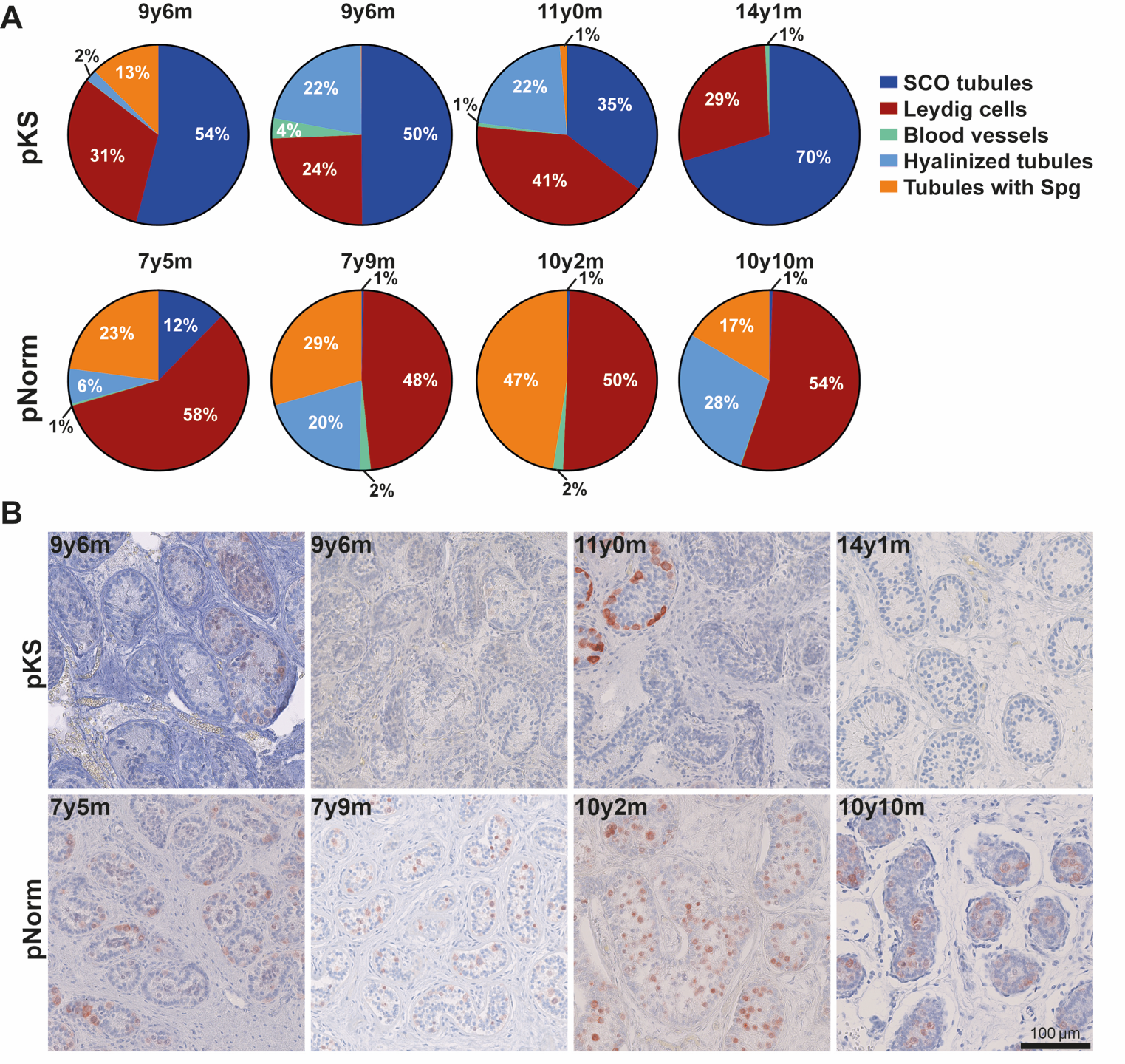
**

**Supplementary Figure S5: Cellularity of the pre-pubertal samples**

**A**: Each testicular biopsy was measured in Adobe Photoshop, and the cellularity was calculated as a percentage of the total area of the sample. Tubules containing spermatogonia (Spg) were evaluated based on MAGE-A4 staining, the rest based on visual inspection. The samples are shown in order according to their age, which is also how they are numbered in the study (e.g. the youngest pre-pubertal Klinefelter syndrome sample 9y6m is also called pKS1 and vice versa for the pre-pubertal normal samples (pNorm)). **B**: MAGE-A4 staining of each sample showing a clear reduction in spermatogonia in the KS samples compared to the controls. Scale bar is 100 µm. Only samples included in RNA‑sequencing final analysis are shown.


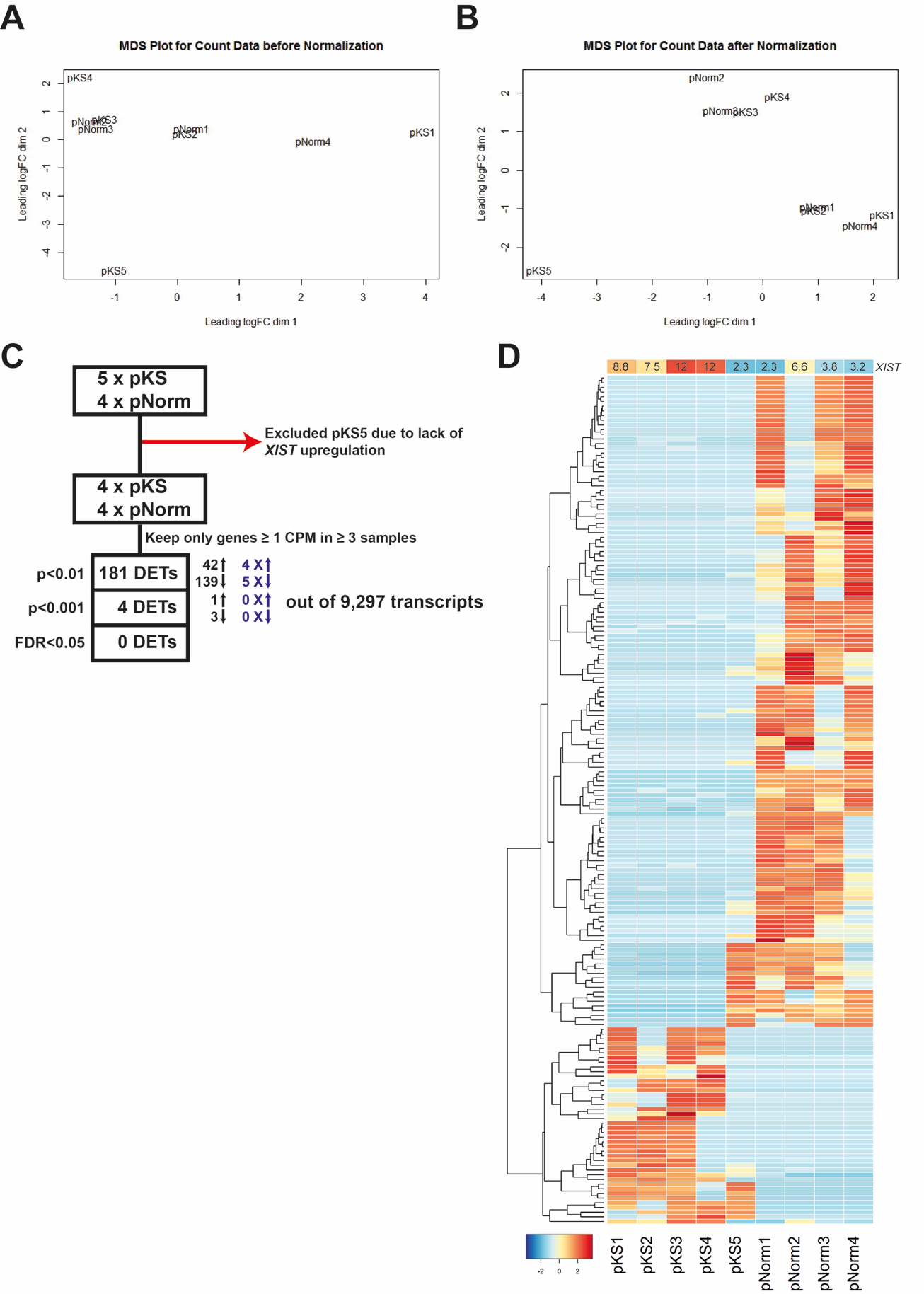


**Supplementary Figure S6: Pre-pubertal initial MDS plots, flowchart and initial heatmap**

Multidimensional scaling (MDS) plot before (**A**) and after (**B**) normalization for library sizes. Notice how the testis sample from the pre‑pubertal Klinefelter syndrome patient no. 5 (pKS5) is completely isolated from the rest of the samples. **C**: Flowchart of the analysis and sample exclusion. **D**: Heatmap of the initially differentially expressed transcripts (DETs). Expression values for *XIST* are shown at higher magnification. Notice no expression of *XIST* in pKS5 and expression in pNorm2.


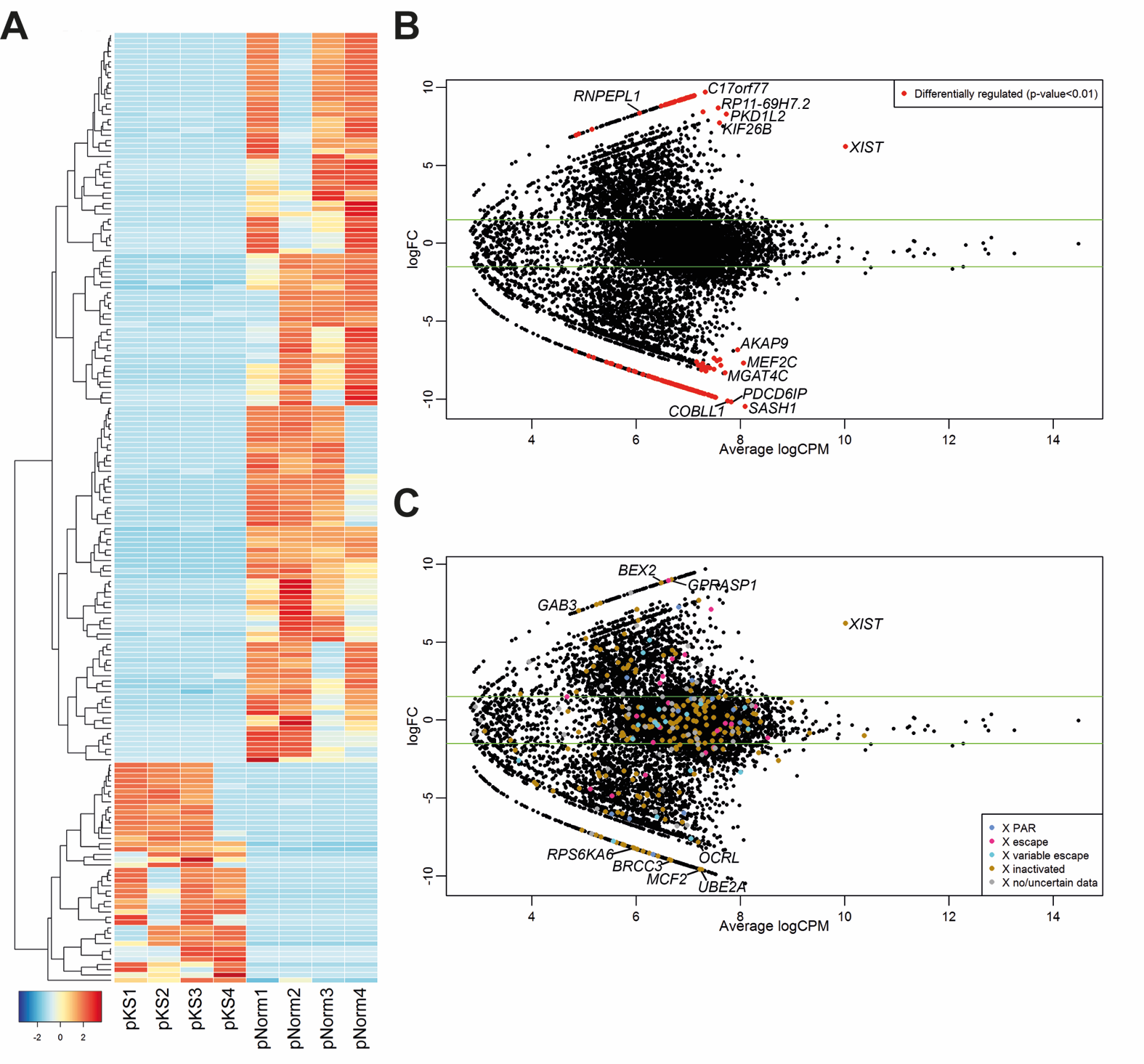


**Supplementary Figure S7: Pre-pubertal differentially expressed transcripts (DETs)**

Using an un-adjusted p-value of 0.01, a total of 181 DETs were identified between pre-pubertal Klinefelter syndrome (pKS) and pre-pubertal controls (pNorm). Of these, 42 were upregulated and 139 were downregulated. **A**: Heatmap of the DETs. **B**: Volcano plot of the DETs. The most significant transcripts are indicated. **C**: X‑chromosomal inactivation status according to (25). The X-chromosomal DETs are indicated.
